# Antennal transcriptome analysis and expression profiles of olfactory genes in *Anoplophora chinensis*

**DOI:** 10.1038/s41598-017-15425-2

**Published:** 2017-11-13

**Authors:** Jingzhen Wang, Ping Hu, Peng Gao, Jing Tao, Youqing Luo

**Affiliations:** Key Laboratory for Silviculture and Conservation of Ministry of Education, Beijing Forestry University, Beijing, 100083 P. R. China

## Abstract

Olfaction in insects is essential for host identification, mating and oviposition, in which olfactory proteins are responsible for chemical signaling. Here, we determined the transcriptomes of male and female adult antennae of *Anoplophora chinensis*, the citrus longhorned beetle. Among 59,357 unigenes in the antennal assembly, we identified 46 odorant-binding proteins, 16 chemosensory proteins (CSPs), 44 odorant receptors, 19 gustatory receptors, 23 ionotropic receptors, and 3 sensory neuron membrane proteins. Among CSPs, *AchiCSP10* was predominantly expressed in antennae (compared with legs or maxillary palps), at a significantly higher level in males than in females, suggesting that *AchiCSP10* has a role in reception of female sex pheromones. Many highly expressed genes encoding CSPs are orthologue genes of *A. chinensis* and *Anoplophora glabripennis*. Notably, *AchiPBP1* and *AchiPBP2* showed 100% and 96% identity with *AglaPBP1* and *AglaPBP2* from *A. glabripennis*, with similar expression profiles in the two species; *PBP2* was highly expressed in male antennae, whereas *PBP1* was expressed in all three tissues in both males and females. These results provide a basis for further studies on the molecular chemoreception mechanisms of *A. chinensis*, and suggest novel targets for control of *A. chinensis*.

## Introduction

*Anoplophora chinensis* (Forster) (Coleoptera: *Cerambycidae*), taxonomically regarded as a synonym of *Anoplophora malasiaca*, is an important worldwide quarantine pest, which is native to East Asia, but which has also been introduced to North America and Europe^1,2^. Although *A. chinensis* is commonly known as the citrus longhorned beetle, and causes extensive damage to citrus trees in its native oriental area, it is actually a polyphagous xylophage that has been documented on hosts from 40 genera^2^. The major economic loss associated with *A. chinensis* results from damage in fruit-tree plantations, whereas the most commonly infested tree genus is *Acer* ^3^. Multiple strategies are currently used to control *A. chinensis*. Identification of pheromones and plant volatiles that are recognized by *A. chinensis* enables chemical baiting to trap the insects. Behavioral studies suggest that both male and female *A. chinensis* can emit volatiles that induce mate orientation in the opposite sex^4,5^, and the contact sex pheromone produced by females can trigger a series of male mating behaviors when it is perceived by the male antennae^6,7^. An important component of the male attractant pheromone of *A. chinensis* is 4-(n-heptyloxy)butan-1-ol^5^. Currently, semiochemically baited traps are being developed to specifically target the Asian long-horned beetle (*Anoplophora glabripennis*)^8^, which also has the potential to greatly improve the sensitivity, reliability, and efficiency of detection of populations of *A. chinensis*.

In insects, olfaction has several vital roles for survival and reproduction, including food selection, mate recognition, location of oviposition sites and predator avoidance^9^. The olfactory proteins involved in recognition of pheromones and odorants include two binding-protein families (odorant-binding proteins (OBPs) and chemosensory proteins (CSPs)), three receptor families (odorant receptors (ORs), ionotropic receptors (IRs), and gustatory receptors (GRs)), sensory neuron membrane proteins (SNMPs), and even enzymes (pheromone-degrading enzymes (PDEs) and odorant-degrading enzymes (ODEs))^9,10^. OBPs and CSPs, which are abundant in the lymph of antennal sensilla, are involved in the first step of the recognition of chemical signals. OBPs are small, soluble proteins with a pattern of six conserved cysteine residues^11^; they are mainly expressed in the antennae in both sexes, and can bind to hydrophobic molecules from the environment and deliver them to the receptors that are located on the membranes of olfactory sensory neurons (OSNs)^12–15^. Pheromone-binding proteins (PBPs) and general odorant-binding proteins (GOBPs) are two subclasses of OBPs. PBPs are considered to be involved in recognition of sex pheromones^16,17^; however, some PBPs cannot distinguish between pheromones and non-pheromones, e.g., *Slit*PBP of *Spodoptera litura* (Fabricius)^18^. Evidence that PBPs specifically bind to a single pheromone component remains elusive; currently, only two PBPs from *Batocera horsfieldi* ^19^ and two from *A. glabripennis* have been identified from transcriptomic sequence data for species in the family *Cerambycidae* ^10^. CSPs are characterized by the presence of four cysteine residues, have a low molecular weight (10–16 kDa), are widely expressed in chemosensory organs and non-olfactory organs in insects, and have multiple functions in chemoreception, growth, and development^20–22^.

Chemosensory reception in insects involves two types of olfactory receptor (ORs and IRs), and one type of GR. ORs are seven-transmembrane-domain proteins that detect volatiles and trigger the transduction of chemical signals to electrical signals that are transmitted to the brain^23,24^. The nonconventional OR previously referred to as OR83b in *Drosophila melanogaster*, OR2 in *Bombyx mori*, and OR7 in mosquitoes was recently renamed Orco, which can selectively distinguish between similar odorant chemical structures owing to the selectivity of the highly conserved and insect-specific Orco and ORs^25^. In addition, *Bmor*OR1 and *Bmor*OR3 expressed in cells located beneath the pheromone-sensitive long sensilla trichodea are pheromone receptor (PR) genes identified in *B. mori* ^26^. IRs are a conserved family with a ligand-gated ion-channel domain that evolved from ionotropic glutamate receptors. Mutations in IR84a, IR64a, IR8a, and IR25a of *Drosophila* are essential for inhibiting odor-evoked neuronal responses^27^. GRs also have a seven-transmembrane-domain topology, and are mainly expressed in the gustatory organs, such as the mouthparts. Multiple GRs have been implicated in the detection of sweet and bitter tastes and CO_2_ vapors^28,29^.

In addition, SNMPs are homologous with the CD36 family, and are membrane proteins that are located in the insect olfactory neurons. To date, two SNMP subfamilies, SNMP1 and SNMP2, have been identified in insects. SNMPs are thought to mediate ligand delivery to receptors, and are expressed late in adult development^30,31^.

Substantial developments have been achieved in the understanding of odorant detection in insects, providing new prospects for integrated pest management, although information about coleopterous olfaction is sparse, and few olfactory gene families have been reported^32–34^.

In this study, we sequenced antennal transcriptomes to identify chemosensory genes in *A. chinensis*, and used quantitative real-time PCR to assess the expression of all CSP genes and two PBP genes in different tissues of both male and female adults. On the basis of these results, we discuss here the potential functions of the products of these genes in olfactory or other physiological processes.

## Results

### Transcriptome sequencing and unigene assembly

The antennal cDNA libraries of male and female *A. chinensis* were sequenced using the Illumina HiSeq. 2500 system. A total of 27,706,136 raw reads were produced from male antennae samples, and the Q20 and Q30 base call accuracies were 93.50% and 86.38%, respectively. A total of 24,274,769 raw reads were produced from female antennae samples, and the Q20 and Q30 base call accuracies were 92.65% and 86.04%, respectively (supplementary Table S1). After trimming the adaptor sequences and removing low-quality sequences, blending male and female sequences, splicing, and assembly (using Trinity), we obtained 59,357 unigenes, with an N50 of 1413 bp and a mean length of 673 bp (supplementary Figure S1). The raw reads for *A. chinensis* have been deposited in the NCBI SRA database (GenBank accession number SRP116677).

### Homology analysis and Gene Ontology (GO) annotation

In total, 25,670 unigenes (43.25% of all 59,357 unigenes) were annotated to at least one of the databases using the BLASTx and BLASTn programs with an E-value cut-off of 10 e-^5^. A total of 17,717 (29.85%), 17,157 (28.90%), 15,824 (26.66%), 8,210 (13.83%), 10,113 (17.04%), 8,646 (14.57%), 24,261 (40.87%), and 9,266 (15.61%) unigenes from *A. chinensis* were annotated using the Nr, Pfam, KOG, COG, Swiss-Prot, KEGG, eggNOG, and GO databases, respectively (supplementary Table S2). Homology searches against the Nr database showed that *A. chinensis* antennal transcriptomes shared the highest homology (55%) with sequences from *Tribolium castaneum*, followed by *Dendroctonus ponderosae* (16%) and *Acyrthosiphon pisum* (2%).

**Figure 1:**
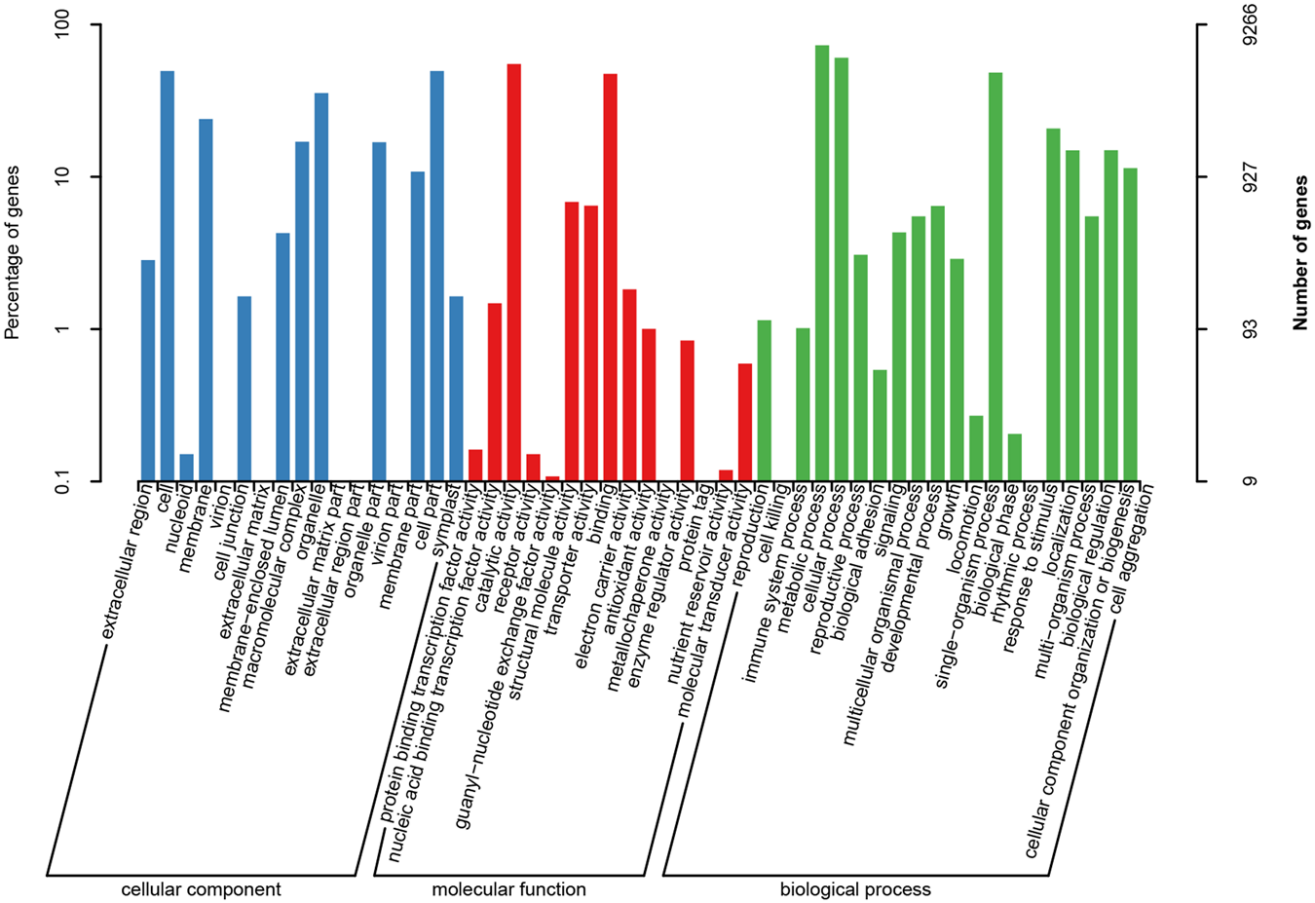
Functional annotation of all unigenes based on gene ontology (GO) categorization. GO analysis was performed at the level for three main categories (cellular component, molecular function, and biological process).

GO annotation was obtained with BLAST2GO. Only 9,266 of the 25,670 *A. chinensis* antennal unigenes (36.1%) were assigned to specific GO terms within the top-level categories ‘biological process’, ‘cellular component’, and ‘molecular function’. The most highly represented terms within these categories were ‘metabolic process’, ‘cellular process’, and ‘single-organism process’. In each of the three GO categories, cell aggregation, metallochaperone activity, and virion part were minimum (Fig. 1).

### Identification of putative OBPs

We identified 46 transcripts encoding putative OBPs in *A. chinensis* antennal transcriptomes. Among these genes, 23 had complete open reading frames (ORFs) ≥ 400 bp that included sequences encoding signal peptides. Based on the number and location of the conserved cysteines, 12 of the 23 full-length *Achi*OBPs lacked the conserved cysteines C2 and C5 (see supplementary Figure S2). *AchiOBP3* have additional cysteines located downstream of the conserved C6 residue in addition to the six conserved cysteines (see supplementary Figure S3). Two of these proteins were identified as PBPs (see supplementary Table S3). Remarkably, using BLASTx to analyze gene homology, we found that the sequence identity between *PBP1* from *A. chinensis* (*AchiPBP1*) and *PBP1* from *A. glabripennis* (*AglaPBP1*) is 100%. Almost all of the matching genes encoded OBPs (or PBPs), but *AchiOBP16* showed similarity with the gene from *B. horsfieldi* that encodes Minus-C odorant-binding protein 2. More than one *A. chinensis* gene exhibited a best match with the same species and gene sequence in the database. For example, four unigenes (c13216.graph_c0, c13631.graph_c0, c18867.graph_c0, and c24613.graph_c1) exhibited best matches with *A. glabripennis* ARH65462.1 (see supplementary Table S3).

**Figure 2:**
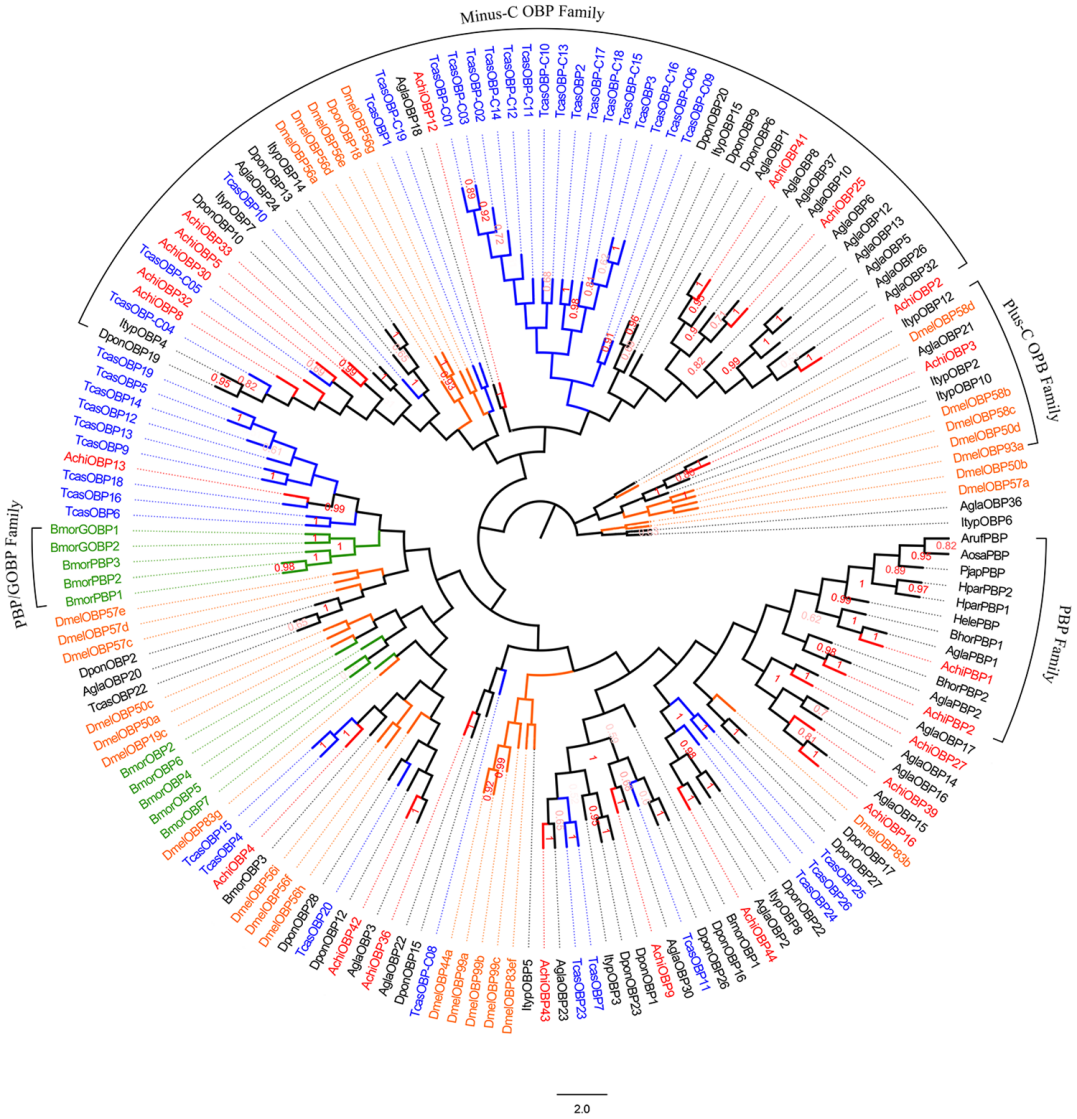
Neighbor-joining phylogenetic tree of candidate odorant-binding proteins (OBPs) The tree was constructed with MEGA5.0 with bootstrap support based on 1,000 replications, and only bootstrap values ≥0.5 are shown at the corresponding nodes. The scale bar represents 2.0 substitutions per site. *A. chinensis* sequences are in red, and the protein names and sequences of the 165 OBPs that were used in this analysis are listed in S8 Table.

The FPKM (Fragments Per Kilobase of transcript per Million mapped reads) values indicated that *AchiOBP11* had the highest expression in the female antennae, whereas *AchiOBP27* had the highest expression in the male antennae. A phylogenetic tree shows the evolutionary relationships between insect OBP genes (Fig. 2). *AchiPBP1* and *AchiPBP2* have high homology with *AglaPBP1* and *AglaPBP2*, respectively. *AchiOBP3*, which encodes a protein with two more cysteine residues than other *A. chinensis* OBPs, clustered with Plus-C OBPs, including those from *Ips typographus*, whereas 9 OBP genes (*AchiOBP2*, *AchiOBP5*, *AchiOBP8*, *AchiOBP12*, *AchiOBP25*, *AchiOBP30*, *AchiOBP32*, *AchiOBP33*, and *AchiOBP41*) that encode proteins lacking two cysteine residues formed part of a Minus-C clade (Fig. 2).

### Identification of putative CSPs

Among 16 putative CSP transcripts, five sequences had full-length ORFs (Table 1). The sequences of 11 *A. chinensis* CSPs had >80% similarity with other species’ CSPs. The FPKM values indicated that *AchiCSP11* had the highest expression in the female antennae, whereas *AchiCSP10* had the highest expression in the male antennae. The *A. chinensis* CSPs were scattered in the phylogenetic tree, and most clustered with *Agla*CSPs (see supplementary Figure S4).

**Table 1:** Best BLASTX matches of *A. chinensis* putative chemosensory protein (CSP) genes.

Number	Gene ID	Unigene length (bp)	ORF length (aa)	Complete ORF	Signal peptide AA	FPKM		Best BLASTX match					
						F	M	Name	Acc. number	Species	Score	E-value	Identity (%)
CSP1	c8588.graph_c0	1177	125	NO	1–17	689.92	136.15	chemosensory protein 1	AGI05161.1	*Dendroctonus ponderosae*	147	1e-39	54
CSP2	c14414.graph_c0	955	100	NO	1–22	0.91	0.36	chemosensory protein 4	AIX97044.1	*Monochamus alternatus*	194	4e-93	93
CSP3	c26429.graph_c0	1163	123	NO	1–18	1.61	0.90	chemosensory protein CSP8	AJO62214.1	*Tenebrio molitor*	187	2e-55	75
CSP4	c39908.graph_c0	713	127	NO	1–19	0.08	0.04	chemosensory protein CSP4	AJO62210.1	*Tenebrio molitor*	147	7e-42	60
CSP5	c28967.graph_c0	1142	115	NO	1–22	7.39	8.99	chemosensory protein 1	AIX97041.1	*Monochamus alternatus*	225	2e-70	92
CSP6	c21469.graph_c0	458	125	YES	1–24	1.14	0.22	chemosensory protein 1	AGI0516.1	*Dendroctonus ponderosae*	160	2e-48	69
CSP7	c26587.graph_c0	1174	149	YES	1–27	72.96	65.88	chemosensory protein 12	AIX97087.1	*Monochamus alternatus*	184	1e-53	87
CSP8	c19256.graph_c0	1491	153	NO	1–19	237.56	560.83	chemosensory protein	AEC04843.1	*Batocera horsfieldi*	169	2e-47	92
CSP9	c13555.graph_c0	591	123	YES	1–18	36.36	38.39	chemosensory protein 10	AIX97085.1	*Monochamus alternatus*	228	4e-74	87
CSP10	c24670.graph_c0	597	126	YES	1–18	67.01	4192.36	chemosensory protein 3	AIX97043.1	*Monochamus alternatus*	202	8e-64	85
CSP11	c13227.graph_c0	845	129	NO	1–18	3405.40	1942.16	chemosensory protein 5	AIX97045.1	*Monochamus alternatus*	232	2e-74	86
CSP12	c29096.graph_c0	2302	138	NO	1–20	31.83	26.11	chemosensory protein 8	AIX97040.1	*Monochamus alternatus*	271	7e-84	96
CSP13	c30244.graph_c0	1328	127	NO	1–16	6.17	1.67	chemosensory protein 9	AIX97084.1	*Monochamus alternatus*	172	3e-49	90
CSP14	c36673.graph_c0	2856	126	NO	1–18	33.09	35.29	chemosensory protein 7	AIX97047.1	*Monochamus alternatus*	199	3e-56	77
CSP15	c34532.graph_c0	2647	309	NO	1–23	12.16	13.48	chemosensory protein 6	AIX97046.1	*Monochamus alternatus*	297	2e-90	87
CSP16	c8520.graph_c0	578	126	YES	1–18	633.96	305.68	chemosensory protein 11	AIX97086.1	*Monochamus alternatus*	233	3e-76	86

### Identification of putative ORs

**Figure 3:**
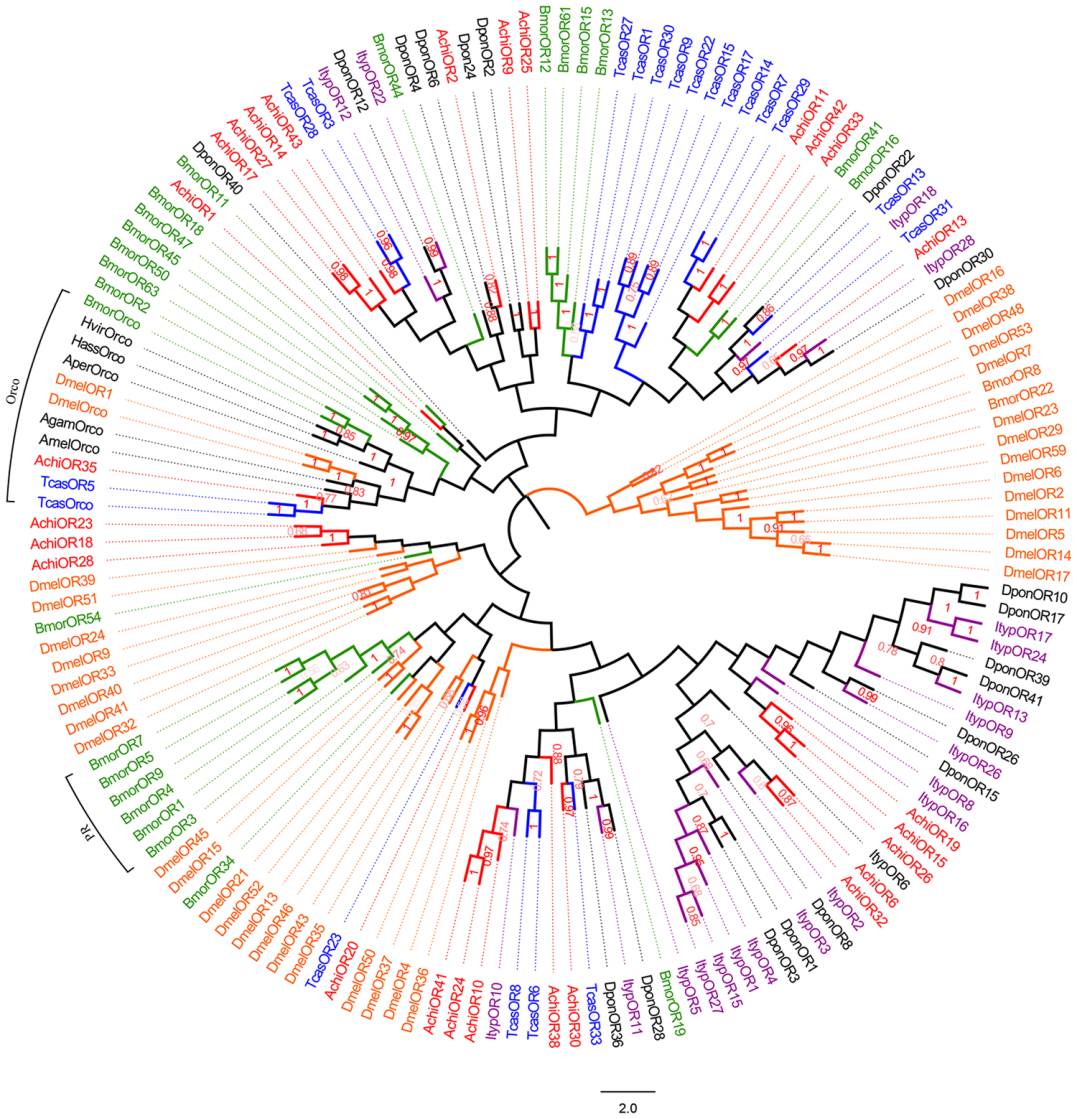
Neighbor-joining phylogenetic tree of candidate odorant receptors (ORs) The tree was constructed with MEGA5.0 with bootstrap support based on 1,000 replications, and only bootstrap values ≥ 0.5 are shown at the corresponding nodes. The scale bar represents 2.0 substitutions per site. *A. chinensis* sequences are in red, and the protein names and sequences of the 155 ORs that were used in this analysis are listed in S8 Table.

Among 44 putative OR genes identified in the *A. chinensis* antennal transcriptomes, 10 unigenes had full-length ORFs encoding proteins longer than 328 amino acids (see supplementary Table S4). Three of the sequences (*AchiOR11*, *AchiOR39*, and *AchiOR42*) encoded seven-transmembrane-domain proteins. *AchiOR10*, *AchiOR24*, and *AchiOR41* all matched *Colaphellus bowringi* ALR72551.1, and *AchiOR9*, *AchiOR22*, and *AchiOR25* all matched *C. bowringi* ALR72569.1. Notably, *AchiOR35* had 97% similarity with *M. alternatus* AIX97092.1, and had the highest level of expression in antennae (both male and female), according to FPKM. In a phylogenetic tree, *AchiOR35* clustered in the Orco clade (Fig. 3).

### Identification of putative IRs

**Figure 4:**
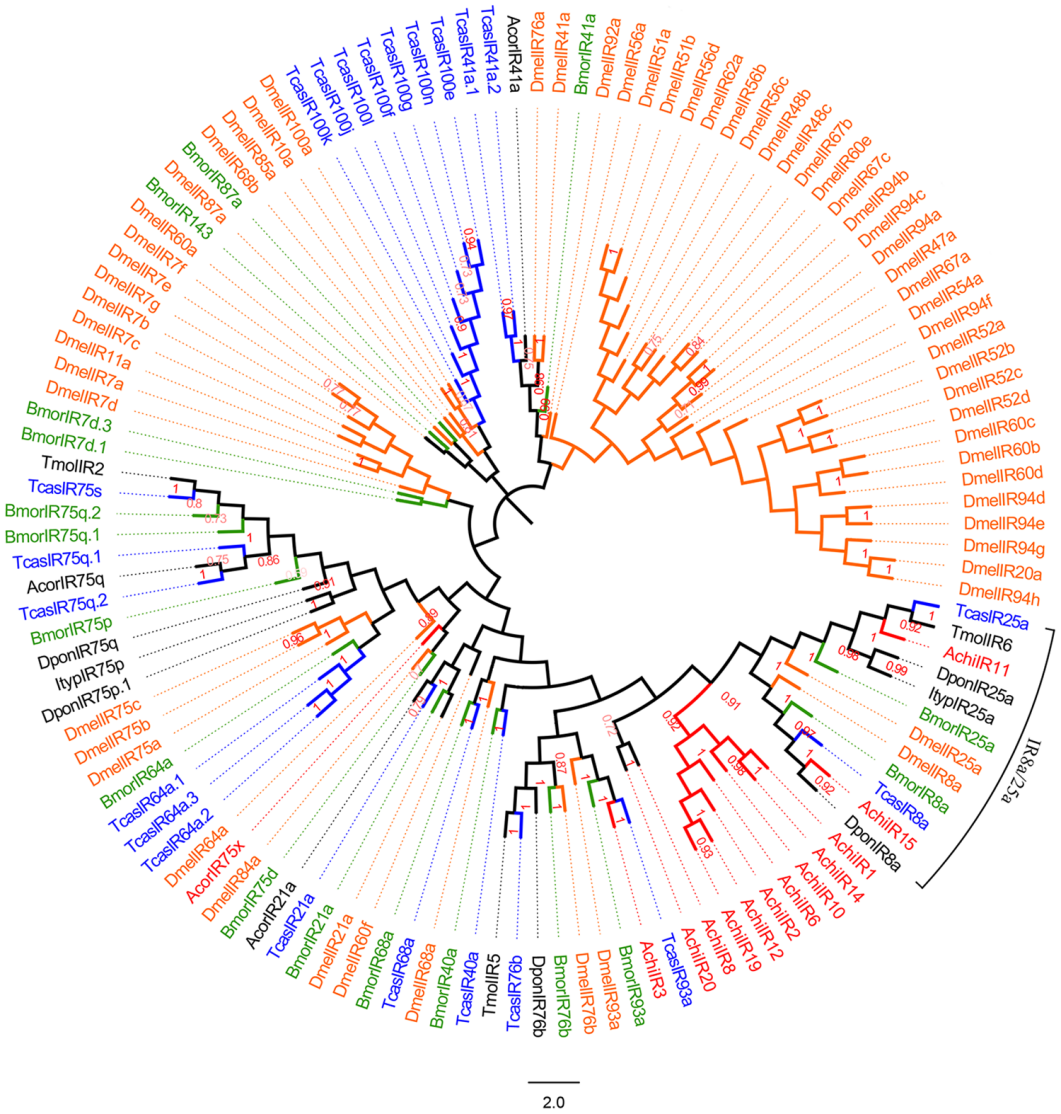
Neighbor-joining phylogenetic tree of candidate ionotropic receptors (IRs). The tree was constructed with MEGA5.0 with bootstrap support based on 1,000 replications, and only bootstrap values ≥ 0.5 are shown at the corresponding nodes. The scale bar represents 2.0 substitutions per site. *A. chinensis* sequences are in red, and the protein names and sequences of the 125 IRs that were used in this analysis are listed in S8 Table.

Among 23 putative IR genes identified in the antennal transcriptomes, four (*AchiIR6*, *AchiIR9*, *AchiIR10*, and *AchiIR19*) had complete ORFs encoding ≥567 amino acids, indicating they were intact full-length unigenes (see supplementary Table S5). The BLASTX searches indicated that *AchiIR4*, *AchiIR5*, and *AchiIR10* all matched *Phyllotreta striolata* ANQ46493.1. The FPKM values indicated that *AchiIR10* had the highest expression in both male and female antennae, whereas there was no identifiable expression of *AchiIR18* or *AchiIR22* in female antennae. The IR phylogenetic tree showed segregation between *D. melanogaster* genes and others. *Achi*IR15 clustered with IR8a sequences from other insects, and *AchiIR11* clustered with *IR25a* sequences (Fig. 4).

### Identification of putative GRs

**Figure 5:**
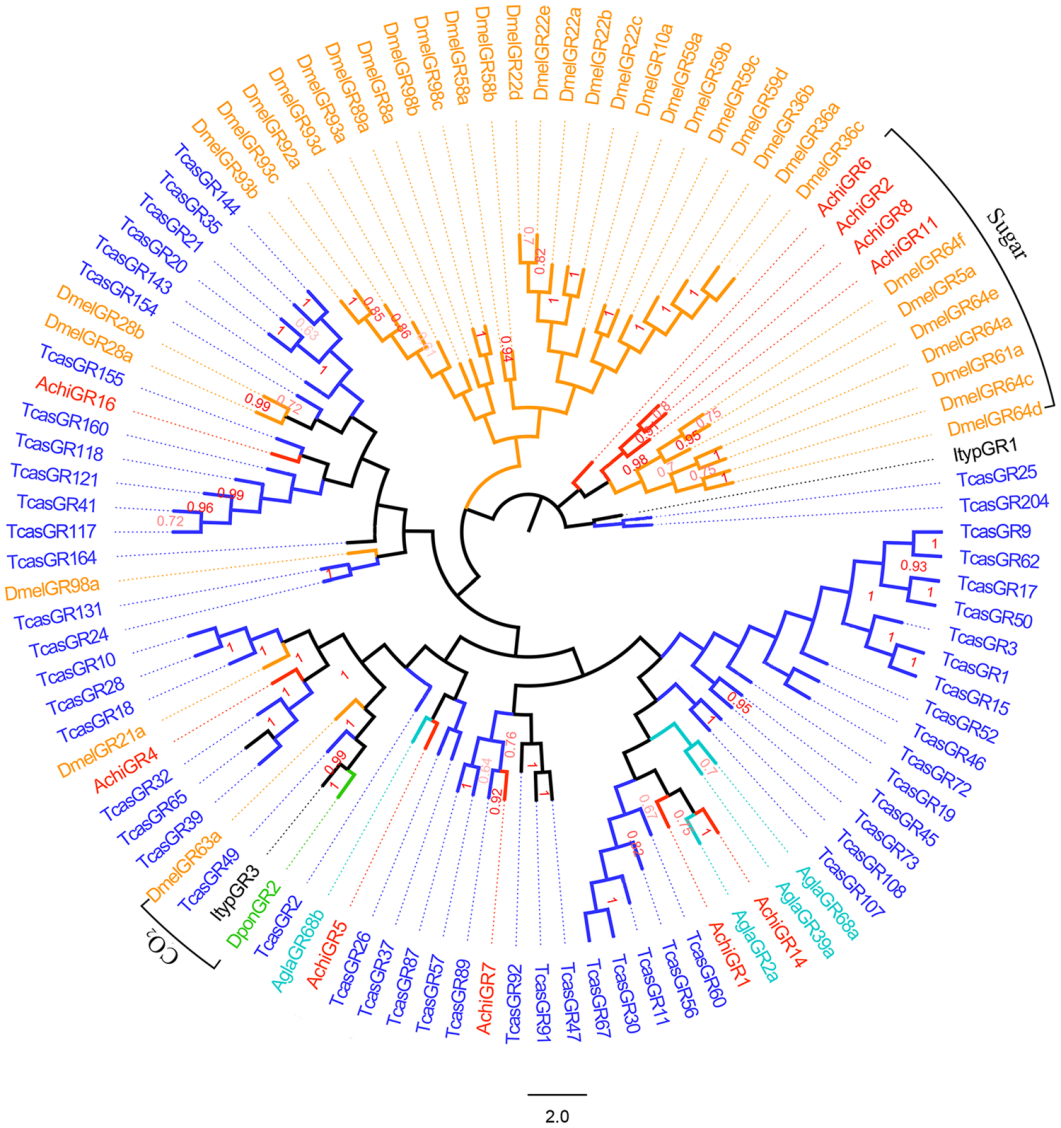
Neighbor-joining phylogenetic tree of candidate gustatory receptors (GRs). The tree was constructed with MEGA5.0 with bootstrap support based on 1,000 replications, and only bootstrap values ≥ 0.5 are shown at the corresponding nodes. The scale bar represents 2.0 substitutions per site. *A. chinensis* sequences are in red, and the protein names and sequences of the 106 GRs that were used in this analysis are listed in S8 Table.

Among 19 putative GR genes, four (*AchiGR2*, *AchiGR6*, *AchiGR11*, and *AchiGR19*) had complete ORFs encoding ≥357 amino acids, indicating they were almost full-length genes (see supplementary Table S6). *AchiGR2* and *AchiGR11* encoded seven-transmembrane-domain proteins. All of the *AchiGRs* had low levels of expression in the antennae of both females and males. A phylogenetic analysis suggested that the highly similar *AchiGR2*, *AchiGR6*, *AchiGR8*, and *AchiGR11* were members of the sugar-receptor subfamily, whereas *AchiGR4* was related to known CO_2_-receptor genes (Fig. 5).

### Identification of putative SNMPs

**Figure 6:**
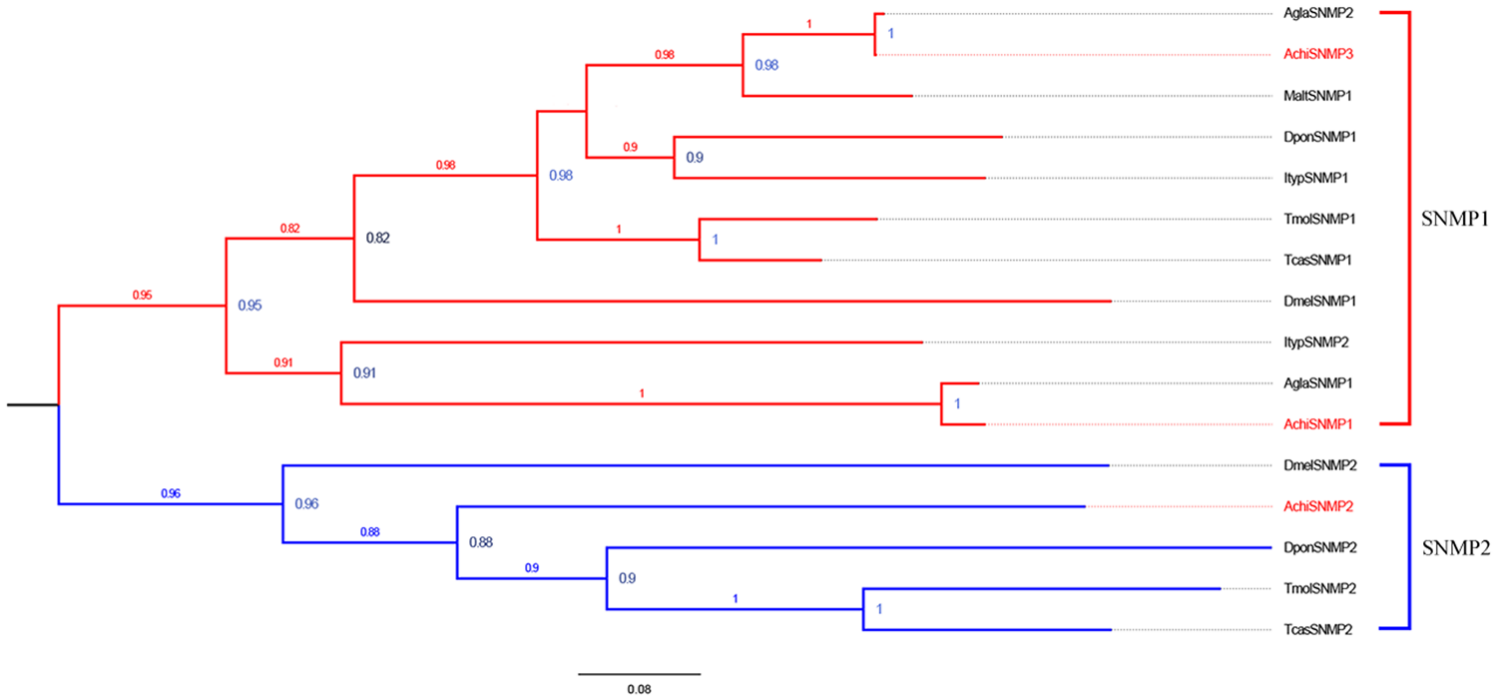
Neighbor-joining phylogenetic tree of candidate sensory neuron membrane proteins (SNMPs). The tree was constructed with MEGA5.0 with bootstrap support based on 1,000 replications, and only bootstrap values ≥ 0.5 are shown at the corresponding nodes. The scale bar represents 0.08 substitutions per site. *A. chinensis* sequences are in red, and the protein names and sequences of the SNMPs that were used in this analysis are listed in S8 Table.

Among three putative *Achi*SNMP genes in the antennal transcriptomes, *Achi*SNMP1 and *Achi*SNMP3 had complete ORFs encoding 529 and 561 amino acids respectively and were full-length genes (see supplementary Table S5). The SNMP genes clustered into two clades, with *AchiSNMP1* and *AchiSNMP3* in the SNMP1 family, and *AchiSNMP2* in the SNMP2 family (Fig. 6).

### Homologous CSPs in A. chinensis and A. glabripennis

**Figure 7:**
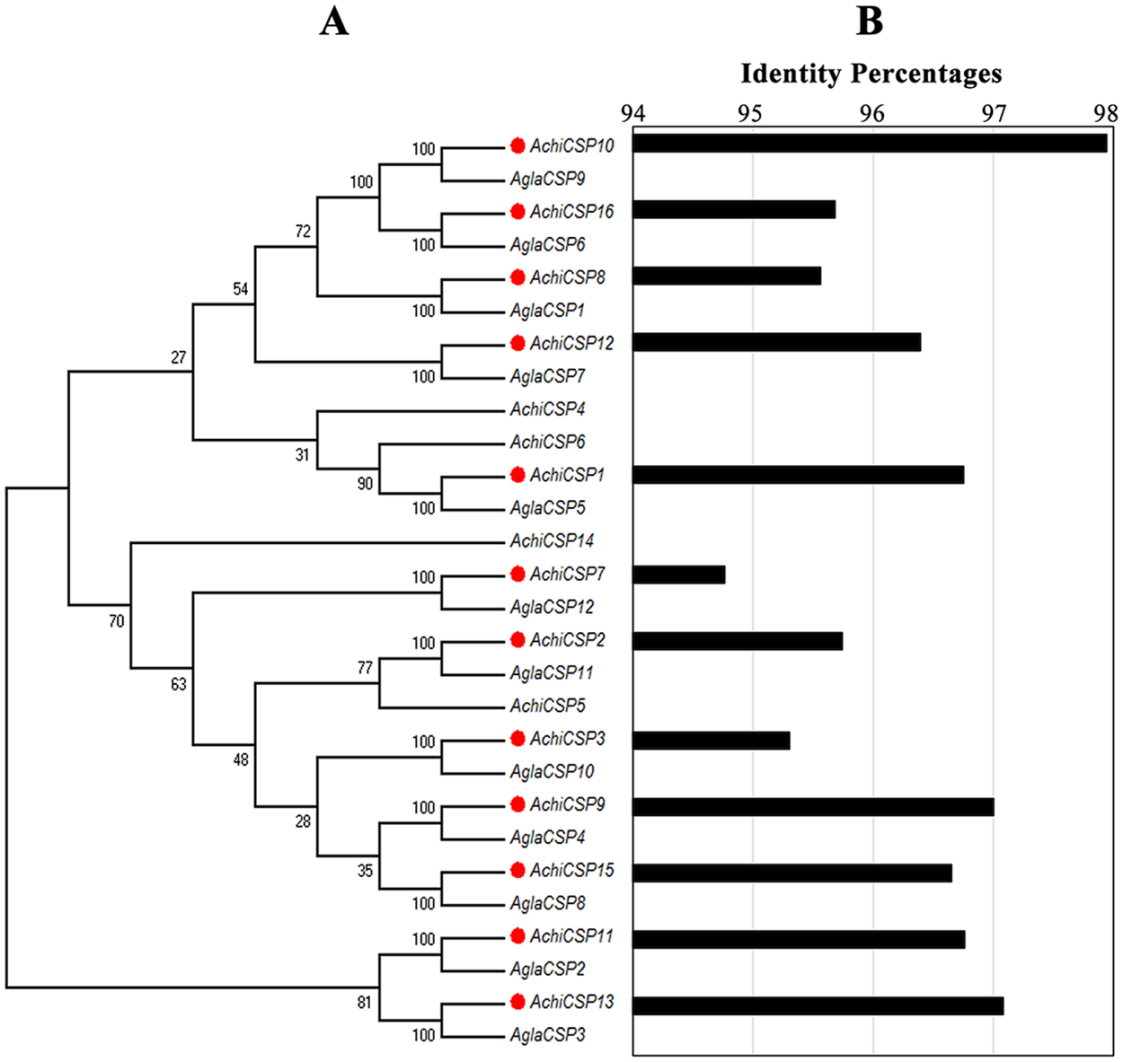
Pairwise comparison of the CSPs from *A. chinensis* and *A. glabripennis*. A: neighbor-joining tree of the CSPs from *A. chinensis* and *A. glabripennis*. The tree was constructed with MEGA5.0 with bootstrap support based on 1,000 replications. B: sequence identity percentages of the homologous CSPs from the two beetles.

Sequence comparison between *A. chinensis* and *A. glabripennis* identified 12 pairs of homologous CSPs whose sequence similarities were higher than 90% (Fig. 7B). Four CSPs (*Achi*CSP4, *Achi*CSP5, *Achi*CSP6, and *Achi*CSP14) were *A. chinensis*-specific (Fig. 7A). The alignment length of CSPs from *A. chinensis* and *A. glabripennis* was longer than 400 bp, with fewer than 25 bp of mismatches (see supplementary Table S7).

### Tissue expression analysis of CSPs and PBPs

**Figure 8:**
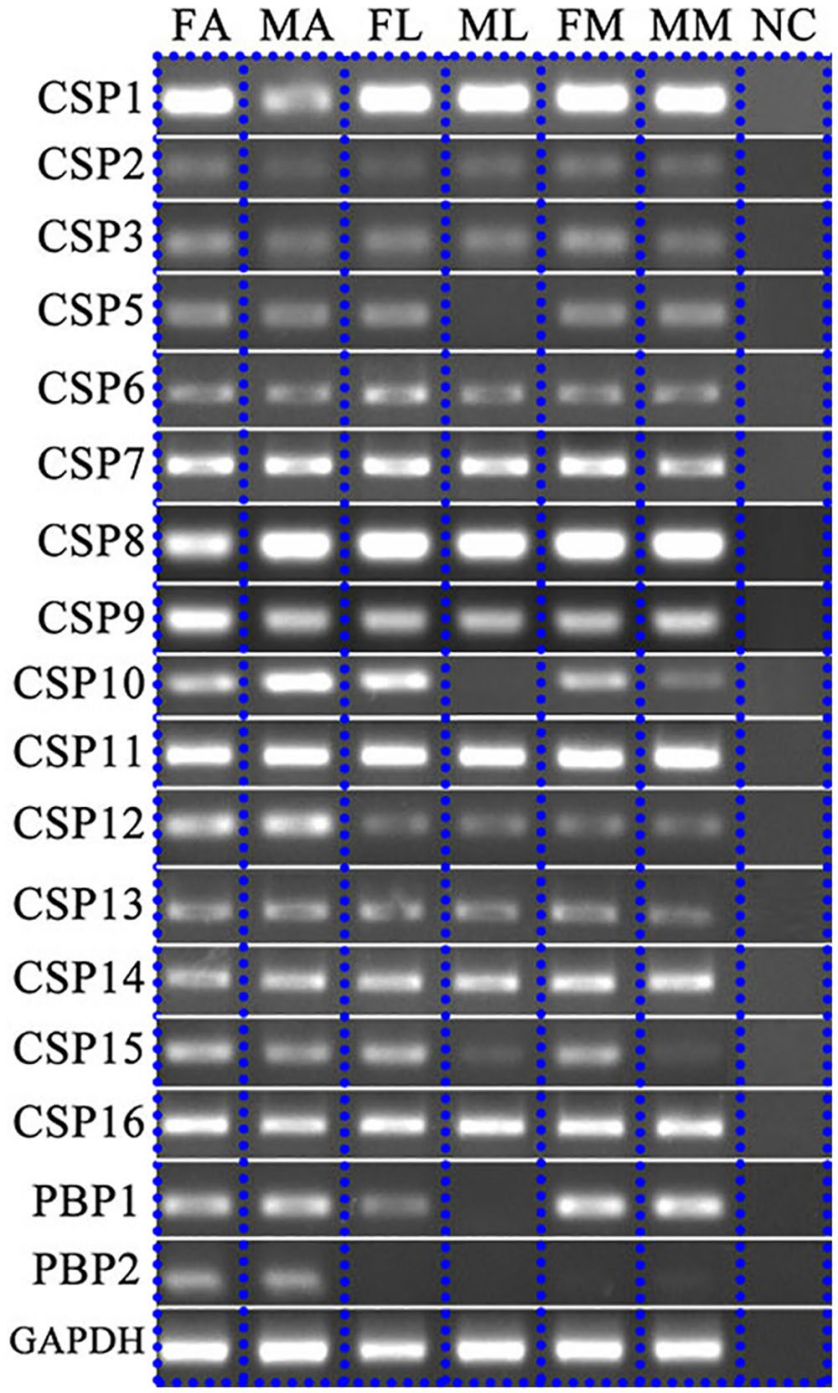
*A. chinensis* CSPs and PBPs transcript levels in different tissues as evaluated by RT-PCR. FA, female antennae; MA, male antennae; FL, female leg; ML, male leg; FM, female maxillary palp; MM, male maxillary palp; NC: no template control. GAPDH used as a reference gene for each cDNA template. the similar intensity of GAPDH bands among different tissues indicates the use of equal template concentrations.

We verified *AchiCSP* and *AchiPBP* expression in antennae and surveyed the expression patterns of 16 *AchiCSPs* and two *AchiPBPs* in three chemosensory tissues (antennae, legs, and maxillary palps) in both females and males using RT-PCR (Fig. 8). The bands of five CSPs (*AchiCSP1*, *AchiCSP*8, *AchiCSP*9, *AchiCSP*11, and *AchiCSP*16) were relatively intense not only in antennae, but also in the other tissues. Although the bands of *AchiCSP*2, *AchiCSP*3, *AchiCSP*4, *AchiCSP*5, and *AchiCSP*13 were very weak or undetectable, this may be due to the relatively low expression levels of these genes, which is consistent with their low abundances (FPKM < 10) (Table 1). PBP2 was only expressed in antennae in both females and males.

**Figure 9:**
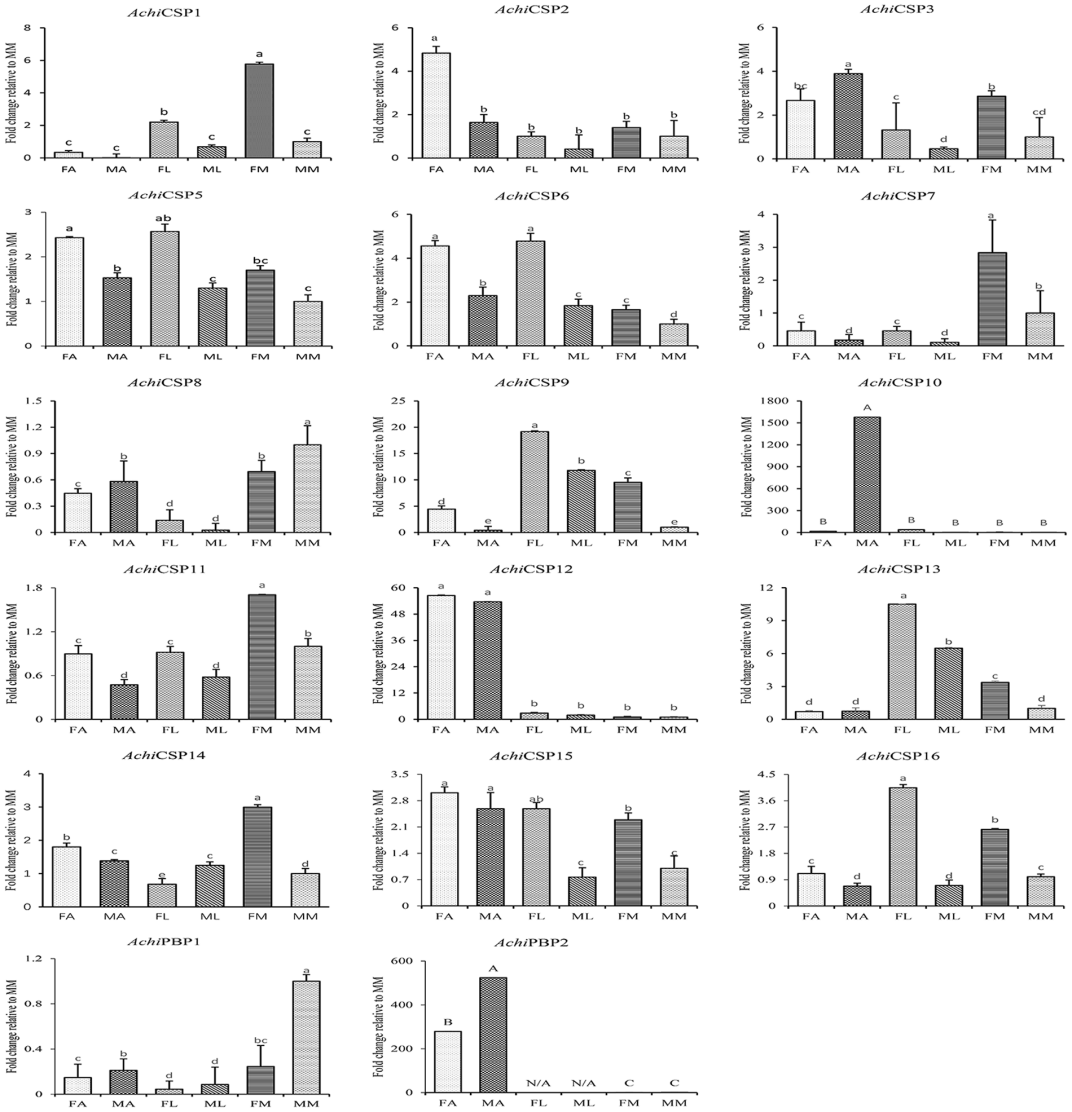
Pheromone-binding protein (PBPs) and Chemosensory protein (CSPs) transcript levels in different tissues of *A. chinensis* FA, female antennae; MA, male antennae; FL, female leg; ML, male leg; FM, female maxillary palp; MM, male maxillary palp. Fold changes are relative to transcript levels in MM. Bars with different letters are significantly different from each other (a, b, c, d, *p* < 0.05; A, B, *p* < 0.01). N/A indicates that the transcript level was too low to measure. NA means the transcript level too low to measure.

To confirm the RT-PCR results, fluorescence quantitative real-time PCR was conducted to determine the expression patterns of CSPs and PBPs in different tissues. In these analyses, expression of all *Achi*CSP genes in the tested tissues was consistent with the RT-PCR results. *AchiCSP10* and *AchiCSP12* had higher expression in the antennae than elsewhere, and *AchiCSP10* expression in the male antennae was highly significantly different from expression in other tissue types. *AchiPBP2* had a significantly higher level of expression in the antennae than in other tissues, and also a significantly higher level of expression in the male antennae than in the female antennae (Fig. 9).

## Discussion

Olfactory genes have been studied in 20 species of Coleoptera to date, but only in three species of *Cerambycidae* (*M. alternatus* ^35^, *B. horsfieldi* ^19^, and *A. glabripennis* ^10^). Here, we identified numerous olfactory genes via sequencing using the Illumina Hiseq™ 2500 platform to analyze the antennal transcriptome of *A. chinensis* as a step toward understanding olfactory processing in this and related species. In the transcriptome sets, we identified 59,357 unigenes with a mean length of 673 bp, a minimum length 201 bp, and a maximum length 10,205 bp, indicating the high quality and depth of sequencing at the transcriptome level. Homology searches against the Nr database showed that *A. chinensis* antennal transcriptomes share high homology with sequences from *T. castaneum* (55%) and *D. ponderosae* (16%), possibly due in part to the identification of many genes, including olfactory genes, in the *T. castaneum* ^32^ and *D. ponderosae* ^36^ genomes.

We identified 46 candidate OBP gene sequences, 16 CSPs, 44 ORs, 19 GRs, 23 IRs, and 3 SNMPs from the antennae of *A. chinensis*. By comparison, 40 OBPs, 12 CSPs, 37 ORs, 11 GRs, 4 IRs, 2 SNMPs, and 1 ODE were previously identified in *A. glabripennis* ^10^. We did not identify any ODE genes, suggesting that they are lowly expressed in *A. chinensis* and that some are species-specific. Functional characterization of these genes from the two species may help to explain host selection. Evidence of the possibility requires us further functional research of two species in future. Our results contribute to the understanding of the vital role of olfactory proteins and olfactory processing in *A. chinensis* and related species.

An OBP typically has from 120 to 150 amino acids and a mass of approximately 14 kDa, including a signal peptide^37^, and 23 putative *A. chinensis* OBPs conformed to these criteria. A phylogenetic tree grouped OBP sequences into several categories. Classic OBPs, which include the PBP-GOBP group, are characterized by a conserved six-cysteine-residue pattern. *Achi*OBP3 encodes a protein with two additional cysteine residues and one proline, making it the Plus-C OBP of *A. chinensis*, which grouped together with the Plus-C OBPs from other insect species. Another 9 *Achi*OBPs were missing two of the conserved cysteines (C2 and C5), and were members of the Minus-C class. The Plus-C and Minus-C subfamilies may contribute to the diverse roles of OBPs in different biological processes^38^. Most of the *Achi*OBPs had a high level of homology with *Agla*OBPs, and as the closely related species *A. chinensis* and *A. glabripennis* have the same host trees, we speculate that these OBPs recognize the same volatiles that are emitted by the hosts. Notably, A*chiPBP1* had 100% identity with *AglaPBP1*, and *AchiPBP2* had 96% identity with *AglaPBP2*, and these genes had the same expression profiles in the two insect species. Both *A. chinensis* and *A. glabripennis* contain 4-(n-heptyloxy) butanol in the attractant pheromones produced by the males, and the high level of similarity between the *Achi*PBPs and *Agla*PBPs suggests that they capture the same pheromones. *AchiPBP2* had a high level of expression in the antennae, suggesting a putative role for this protein in the detection of the female sex pheromones, as with PBPs from *Cydia pomonella* and *Hyphantria cunea* ^39,40^, and as with *AglaPBP2*. *AchiPBP1* was equally expressed in both sexes (similar to *PxylPBP2* of *Plutella xyllotella* ^41^). Female moths are capable of detecting and monitoring both female and male sex pheromones^42,43^. *AchiPBP1* expression in females suggests that it might be associated with recognition of male-produced pheromone. In female *A. glabripennis*, five sex-pheromone components have been identified: (*Z*)-9-tricosene; (*Z*)-9-pentacosene; (*Z*)-7-pentacosene; (*Z*)-9-heptacosene; and (*Z*)-7-heptacosene^44^. In female *A. chinensis*, different sex-pheromone components have been identified: (*Z*)-18-heptacosen-10-one; (18*Z*,21*Z*)-heptacosa-18,21-dien-10-one; and (18*Z*,21*Z*, 24*Z*)-heptacosa-18,21,24-trien-10- one^6,7^. The mechanism by which PBPs with similar structures distinguish a variety of pheromone components is not yet known, and the function of *A. chinensis* PBPs should be studied further to determine their roles in pheromone recognition.

The 16 CSP genes predicted in the *A. chinensis* transcriptome is more than the 12 CSP-encoding genes identified in *A. glabripennis*, but less than the 19 genes in *M. alternatus* and *D. helophoroides* ^45^, or the 20 genes in *T. castaneum* ^32^. In a phylogenetic analysis, all CSPs of *D. melanogaster* were contained in a single clade, whereas those of *A. chinensis* and *A. glabripennis* were divided into several different branches, as has previously been observed for the CSP genes of *Mamestra brassicae* ^10,46^. CSPs are more highly conserved than OBPs, and are widely expressed in different parts of the insect body. *AchiCSP12* clustered with *AglaCSP7*; both genes are highly expressed in the antennae and could be involved in chemoreception. *AchiCSP10* was almost exclusively expressed in male antennae, and similarly biased expression has previously been observed for *SinfCSP19* of *Sesamia inferens* ^47^. Palps are primarily responsible for sensing the trail pheromone in *A. glabripennis* ^48^. Similar to *AglaCSP3*, *AglaCSP6*, *AglaCSP10*, and *AglaCSP12*. expression of *AchiCSP1*, *AchiCSP7*, *AchiCSP11*, and *AchiCSP14* was enriched in maxillary palps, which might be related to the delivery of these semiochemicals. CSPs are likely involved in carrying semiochemicals in the moth *M. brassicae* ^49^. In addition, some CSPs were highly expressed in the legs of *A. chinensis*, and might participate in other physiological processes besides chemoreception in this species. *AchiCSP1*, *AchiCSP8*, *AchiCSP10*, *AchiCSP11*, *AchiCSP16*, and their homologous genes (*AglaCSP5*, *AglaCSP1*, *AglaCSP9*, *AglaCSP2*, and *AglaCSP6*) were highly expressed. We can test the functions of these homologous gene pairs, which avoids the high cost associated with functional analysis of every CSP as well as the bias and incompleteness associated with the random selection of CSPs.

A total of 44 OR genes were identified in the *A. chinensis* antennal transcriptomes, which is similar to the 43 genes in *I. typographus*, fewer than the 49 in *D. ponderosae* ^34^ and the 57 in *M. caryae* ^33^, and considerably fewer than the 110 in *T. castaneum* ^50^, but more than the 37 in *A. glabripennis* ^10^ and the 20 in *Tenebrio molitor* ^17^. In a phylogenetic analysis, *AchiOR35* clustered with Orco genes^51^, particularly those encoding Orco orthologues in *T. castaneum*. *Achi*OR35 could be the Orco in *A. chinensis*. A total of 23 candidate IR genes were identified. Phylogenetic analysis suggests that *Achi*IR15 and *Achi*IR11 are the IR8a and IR25a genes, respectively, of *A. chinensis*. These IR subtypes have essential roles in tuning IR sensory cilia targeting and IR-based sensory channels^52^.

SNMPs belong to two subfamilies, SNMP1 and SNMP2^53,54^. In a phylogenetic analysis, *AchiSNMP1* and *AchiSNMP3* clustered into the SNMP1 subfamily, and *AchiSNMP2* clustered into SNMP2. Members of the insect SNMP1 subfamily are expressed in the pheromone-sensitive ORNs; however, the SNMP2 proteins are expressed in the supporting cells, rather than the ORNs^54^. We also identified 19 GR genes, which is more than the 11 identified in the *A. glabripennis* antennal transcriptome^10^, the six identified in *I. Typographus*, and the two identified in *D. Ponderosae* ^34^. A phylogenetic analysis suggested that *Achi*GR4 might have a role in CO_2_ detection, whereas four *Achi*GRs clustered with *Dmel*GRs that encode sugar receptors. Sugar recognition is thought to be involved in host-plant selection and egg-laying behavior of codling moth (*C. pomonella*) females^55,56^. Further research is required to determine the precise roles of the SNMP and GR proteins in *A. chinensis*.

## Materials and Methods

### Insect and tissue collection

Adult *A. chinensis* (Forster)were collected from host trees (*Melia azedarach* L.) at Fuzhou, Fujian Province, China, and reared in our laboratories at 25 ± 1 °C. Antennae from two males and two females were excised and stored in RNAlater (Ambion, Austin, TX, USA) at −80 °C until RNA extraction. All operations were performed according to ethical guidelines to minimize pain and discomfort to the insects.

### Total RNA extraction, cDNA library construction, and Illumina sequencing

Total RNA was extracted from two female antennae and two male antennae using TRIzol reagent (Ambion) and the RNeasy Plus Mini Kit (No. 74134; Qiagen, Hilden, Germany), following the manufacturers’ instructions. RNA quantity was detected using the NanoDrop 8000 (Thermo, Waltham, MA, USA). RNA of male and female antennae was used to construct the cDNA library respectively. The cDNA library construction and Illumina sequencing of samples were performed at CapitalBio Corporation (Beijing, China). mRNA samples were purified and fragmented using the TruSeq RNA Sample Preparation Kit v2-Set A (No. RS-122-2001; Illumina, San Diego, CA, USA). Random hexamer primers were used to synthesize the first-strand cDNA, followed by synthesis of the second-strand cDNA using buffer, dNTPs, RNase H, and DNA polymerase I at 16 °C for 1 h. and the products were amplified by PCR and quantified precisely using the Qubit DNA Br Assay Kit (Q10211; Invitrogen, Carlsbad, CA, USA). After adenylation of 3′ ends of DNA fragments, NEBNext Adaptor with hairpin loop structure were ligated to prepare for hybridization. In order to select cDNA fragments of preferentially 150 ~ 200 bp in length, the library fragments were purified with AMPure XP system (Beckman Coulter, Beverly, USA). Then 3 μl USER Enzyme (NEB, USA) was used with size-selected, adaptor-ligated cDNA at 37 °C for 15 min followed by 5 min at 95 °C before PCR. Then PCR was performed with Phusion High-Fidelity DNA polymerase, Universal PCR primers and Index (X) Primer. At last, PCR products were purified (AMPure XP system) and library quality was assessed on the HiSeq. 2500 platform.

### Assembly and functional annotation

All raw reads were processed to remove low-quality and adaptor sequences by Trimmomatic (http://www.usadellab.org/cms/index.php?page=trimmomatic), Clean reads assembly was carried out with the short-read assembly program Trinity (Version: r2014-04-13) with the default parameters after combined the male and female clean reads. The largest alternative splicing variants in the Trinity results were called unigenes. The annotation of unigenes was performed by NCBI BLASTx searches against the Nr protein database, with an E-value threshold of 1e-5. The blast results were then imported into the Blast2GO pipeline for GO annotation. The longest ORF of each unigene was determined by the NCBI ORF Finder tool (http://www.ncbi.nlm.nih.gov/gorf/gorf.html). The level of expression of unigenes was indicated using FPKM values, which were calculated by RSEM (RNA-Seq by Expectation-Maximization)^57^.

### Identification of chemosensory genes

Putative unigenes involved in olfaction in *A. chinensis* were confirmed by analysis with the BLASTX program. All candidate OBP, CSP, OR, GR, IR, and SNMP genes were manually checked by tBLASTn in NCBI online.

### Sequence and phylogenetic analysis

Amino acid sequences were deduced by analysis on the ExPASy portal (http://web.expasy.org/translate/). Putative N-terminal signal peptides of candidate OBP and CSP proteins were predicted on the SignalP4.1 Server (http://www.cbs.dtu.dk/services/SignalP/). Transmembrane domains of candidate OR, IR, GR, and SNMP proteins were predicted using the TMHMM server v3.0 (http://www.cbs.dtu.dk/services/TMHMM/). After amino acid sequence alignment using ClustalX (1.83), phylogenetic trees were constructed using the neighbor-joining method in MEGA 5.0 with default settings and 1,000 bootstrap replications^58^, and then the dendrograms were colored and arranged in FigTree v1.4.3. Phylogenetic tree analyses of *Achi*OBPs, *Achi*CSPs, *Achi*ORs, *Achi*IRs, *Achi*GRs, and *Achi*SNMPs were based on the amino acid sequences encoded by the putative chemosensory genes, and on sequences identified from other insects. The protein names and gene accession numbers are indicated in supplementary Table S6.

### Tissue expression analysis of OBP and CSP genes

The expression patterns of CSP and PBP genes in both male and female tissues (antenna, leg, and maxillary palp) were analyzed by were analyzed by RT-PCR and fluorescence quantitative real-time PCR. Antennae, leg and maxillary palps were collected from four adult *A. chinensis* of both male and female and one total RNA was extracted following the methods described above. Equal amounts of RNA from each tissue were used to synthesize cDNA by the PrimeScript RT Reagent Kit with gDNA Eraser (No. RR047A; TaKaRa, Shiga, Japan). Gene-specific primers were designed using Primer3 (http://bioinfo.ut.ee/primer3-0.4.0/) (see supplementary Table S7). The RT-PCR was performed under following conditions: 95 °C for 2 min, followed by 35 cycles of 95 °C for 30 sec, 55 °C for 30 sec, 72 °C for 1 min, and a final extension for 10 min at 72 °C. PCR products were analyzed on 1.5% agarose gel and visualized after staining with ethidium bromide. In additional, the PCR products were selected and verified by DNA sequencing. To reach reproducibility, each sample was examined at least three times with three biological samples.

qPCR analysis was performed on the Bio-Rad CFX96 PCR System (Bio-Rad, Hercules, CA, USA). GAPDH from *A. chinensis* was used as a reference gene. each PCR reaction (25 µl) contained 12.5 µl of SYBR Premix Ex Taq II, 1 µl of each primer (10 mM), 2 µl of sample cDNA (2.5 ng of RNA), and 8.5 µl of dH_2_O (sterile distilled water). qPCR cycling parameters were 95 °C for 30 s, followed by 40 cycles of 95 °C for 5 s and 60 °C for 30 s, and a final gradient from 65 to 95 °C in increments of 0.5 °C for 5 s each, to generate the melting curves. Negative controls without a template were included in each experiment. To examine reproducibility, each qPCR reaction for each tissue was performed in three technical replicates and three biological replicates. Bio-Rad CFX Manager (version 3.1.1517.0823) was used to normalize expression based on ΔΔCq values, with male maxillary palps in analyze mode as control samples, with relative expression calculated by the 2^−ΔΔCT^ method(the amplification efficiency for 17 genes was equal to 100%)^59^. The comparative analyses for every gene among six tissue types were assessed by a one-way nested analysis of variance (ANOVA), followed by Tukey’s honestly significance difference (HSD) tests implemented in SPSS Statistics 18.0. Values are presented as means ± SE.

### Availability of data and materials

All supporting data is included within the article and its additional files. The raw reads for *A. chinensis* were submitted in the NCBI SRA database under the GenBank accession number SRP116677; the entire transcriptome assembly were deposited in the NCBI TSA database under the GenBank accession number GFXC00000000; the olfactory protein gene sequences were submitted to Genbank, accession number is MF975370-MF975415, MF975416-MF975431, MF975432-MF975474, MF975475-MF975492, MF975493-MF975514 and MF975515-MF975517.

### Ethics approval and consent to participate

The citrus longhorned beetle *Anoplophora chinensis* (Coleoptera: Cerambycidae) is an important worldwide quarantine pest in China, which collections were made with the direct permission of Fujian forestry bureau. It’s not included in the “List of Endangered and Protected Animals in China”. All operations were performed according to ethical guidelines in order to minimize pain and discomfort to the insects.

### Authors’ information

Jingzhen Wang: master candidate; major: forest protection; study direction: insect molecular biology. Ping Hu: PhD candidate; major: forest protection; study direction: insect molecular biology and insect chemical ecology. Peng Gao: PhD candidate; major: forest protection; study direction: insect molecular biology and insect chemical ecology. Jing Tao: PhD, study direction: insect molecular biology. Youqing Luo: The Yangtze River Scholar Professor, study direction: Pest Control and forest ecology.

## Electronic supplementary material

Supplementary file
