# Supplementary material for: Antennal transcriptome analysis and expression profiles of olfactory genes in Anoplophora chinensis
Source: Sci Rep. 2017 Nov 13;7:15470. doi: 10.1038/s41598-017-15425-2 (PMC5684370; doi:10.1038/s41598-017-15425-2)
Supplement: Supplementary file 1 — Supplementary file [file 41598_2017_15425_MOESM1_ESM.pdf]

Antennal transcriptome analysis and expression profiles of olfactory genes in *Anoplophora chinensis*

Jingzhen Wang[a], **Ping Hu**[a], **Peng Gao**[a], **Jing Tao**[a*] **and Youqing Luo**[a*]

*Corresponding author

E-mail addresses:

Jingzhen Wang: wjz0707@bifu.edu.cn

Ping Hu: hupingcs@163.com

Peng Gao: gaopeng19900123@bjfu.edu.cn

Jing Tao: taojing1029@hotmail.com

Youqing Luo: youqingluo@126.com

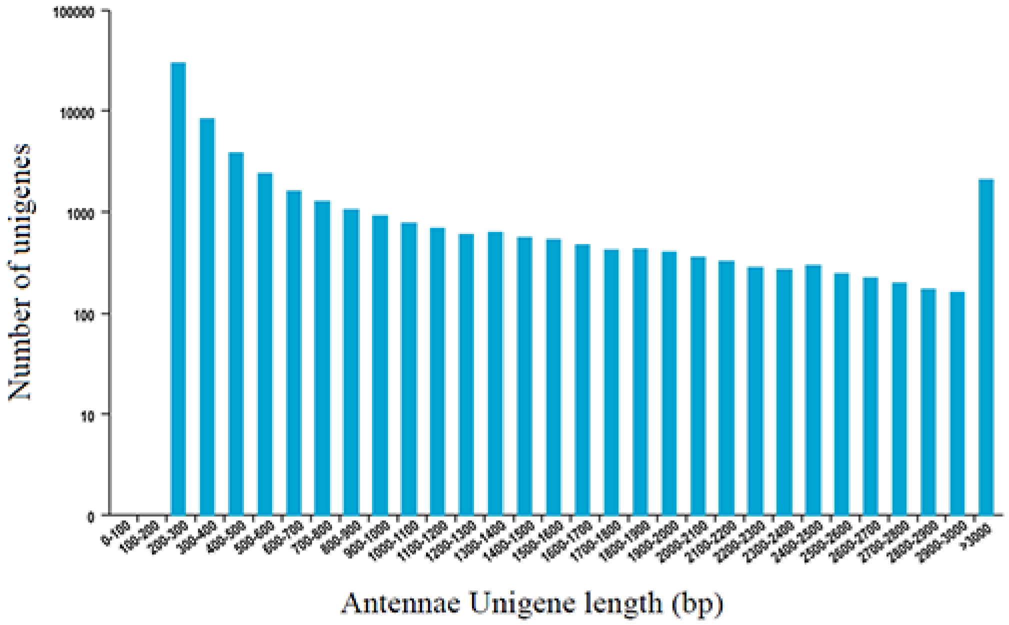

supplementary Figure S1. The size distribution of the unigenes from *A.chinensis* antennal transcriptome

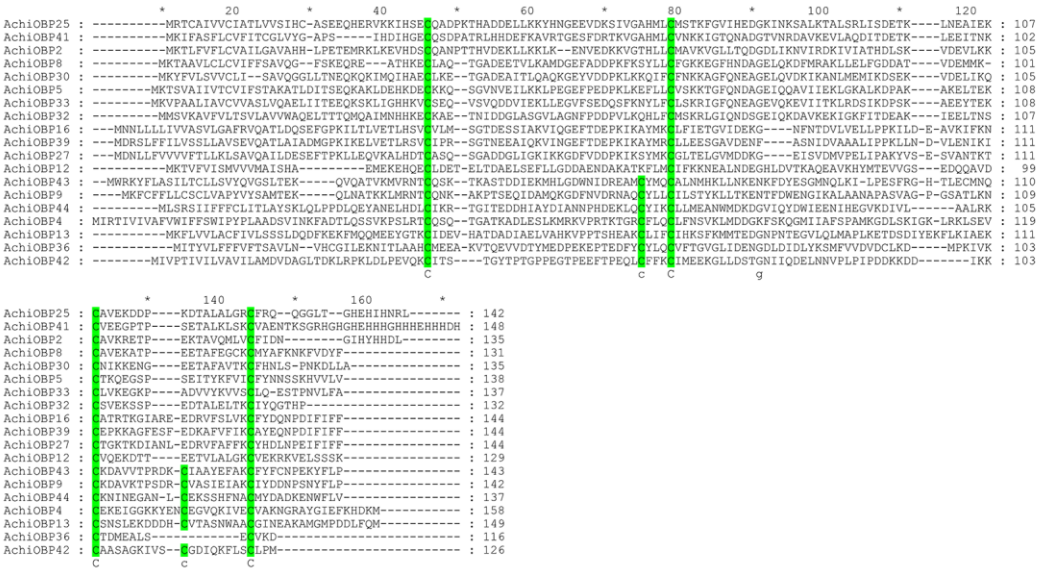

supplementary Figure S2. Alignment of the *A. chinensis* OBPs.

Full-length amino acid sequences of *A. chinensis* OBPs (except *Achi*OBP3) are aligned by ClustalX 1.83 and edited using Genodoc. Green boxes show conserved cysteine residues.

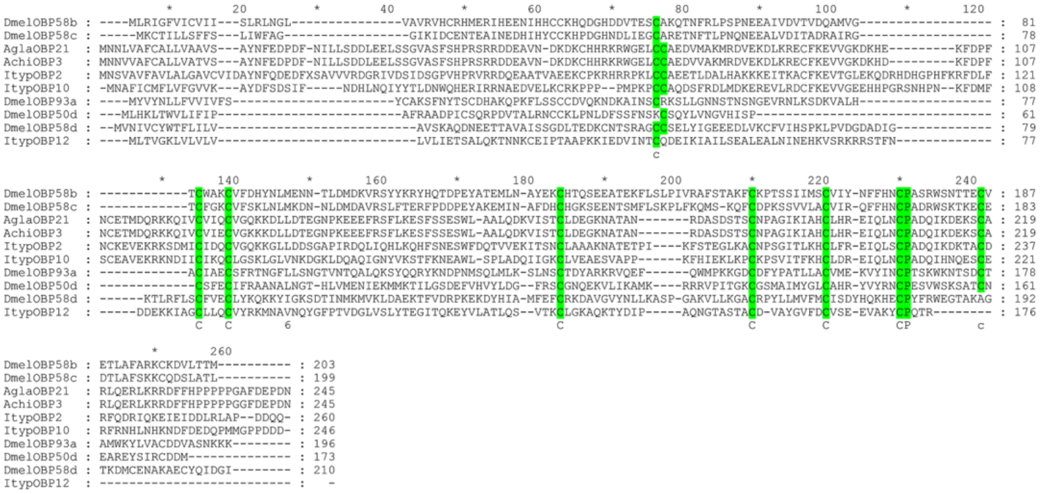

supplementary Figure S3. Alignment of the Plus-C OBPs (*Achi*OBP3) with those of other insect species.

Full-length amino acid sequences are aligned by ClustalX 1.83 and edited using Genodoc. Green boxes show conserved cysteine residues. Accession numbers for all the Plus-C OBPs are listed supplementary Table S6.

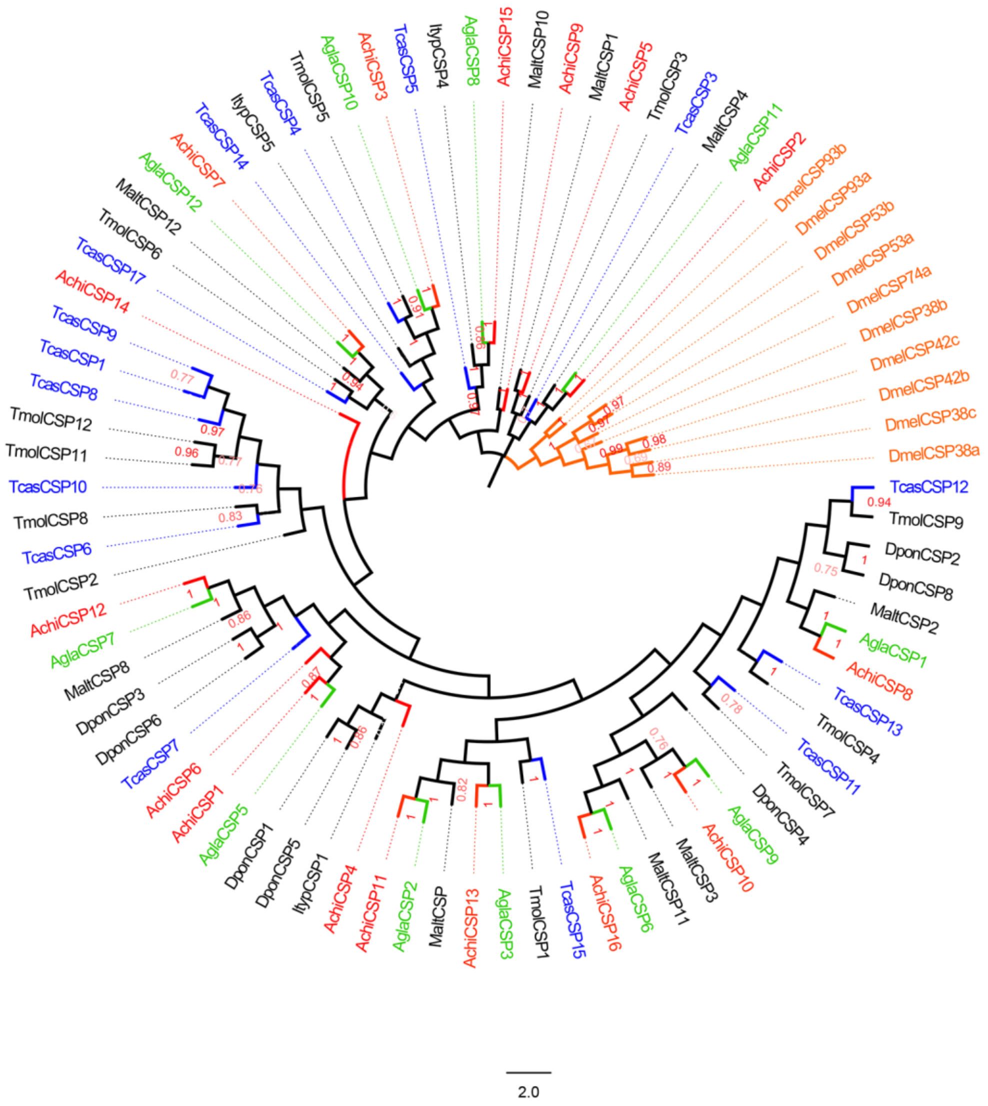

supplementary Figure S4. Neighbor-joining phylogenetic tree of candidate chemosensory proteins (CSPs).

The tree was constructed with MEGA5.0 with bootstrap support based on 1,000 replications, and only bootstrap values ⩾ 0.5 are shown at the corresponding nodes. The scale bar represents 2.0 substitutions per site. *A. chinensis* sequences are in red, and the protein names and sequences of the CSPs that were used in this analysis are listed in supplementary Table S1.

supplementary Table S1. An overview of the sequencing and assembly process.

Ittem	Male	Female
Total Raw Reads	27,706,136	24,274,769
Total Base	8,172,797,452	7,154,388,586
GC Content (%)	42.65	43.07
Raw Reads Q30 (%)	86.38	85.04
Raw Reads Q20 (%)	93.5	92.65
Combined Trinity assembly of the male and female		
Total Number	59,357	
Total Length	39,962,178	
Unigene N50	1,413	
Mean Length	673	

supplementary Table S2. The result of unigenes annotion.

Anno_Database	Annotated Number	300<=length<1000	length>=1000
COG_Annotation	8210	2235	3185
GO_Annotation	9266	2909	1959
KEGG_Annotation	8646	2486	3250
KOG_Annotation	15824	4551	6400
Pfam_Annotation	17157	5285	7230
Swissprot_Annotation	10113	3027	3563
eggNOG_Annotation	24261	7538	8140
nr_Annotation	17717	5041	6102
All_Annotated	25670	8056	8278

supplementary Table S3. Best BLASTX matches of *A. chinensis* putative OBP genes.

Number	Gene ID	Unigene Length (bp)	ORF Length (AA)	Complete ORF	signal peptide AA	FPKM		Blastx match					
						F	M	Name	Acc. number	Species	Score	E-value	Identity (%)
PBP1	c27344.graph_c0	818	136	YES	1-21	57.37	60.99	pheromone binding protein 1	ASA46120.1	*Anoplophora glabripennis*	259	3e-85	100
PBP2	c25366.graph_c0	753	135	YES	1-19	19.49	23.47	pheromone binding protein 2	ASA46121.1	*Anoplophora glabripennis*	226	2e-72	96
OBP1	c13216.graph_c0	502	41	NO	ND	2418.20	452.63	odorant binding protein 7	ARH65462.1	*Anoplophora glabripennis*	179	4e-55	64
OBP2	c37389.graph_c0	565	135	YES	1-17	181.69	13.97	odorant-binding protein 1	APC94199.1	*Pyrrhalta maculicollis*	119	2e-31	50
OBP3	c27143.graph_c0	925	245	YES	1-17	26.63	17.17	odorant-binding protein 24	AIX97039.1	*Monochamus alternatus*	276	1e-90	88
OBP4	c37661.graph_c0	611	158	YES	1-24	0.95	1.06	odorant binding protein 9	ALR72497.1	*Colaphellus bowringi*	174	2e-52	53
OBP5	c19493.graph_c0	682	138	YES	1-19	52.93	136.63	odorant-binding protein 27	AIX97089.1	*Monochamus alternatus*	214	5e-68	78
OBP6	c13631.graph_c0	221	35	YES	ND	12.12	0.67	odorant binding protein 7	ARH65462.1	*Anoplophora glabripennis*	84.7	2e-19	53
OBP7	c30577.graph_c0	1297	166	NO	1-20	54.58	87.60	odorant-binding protein 14	AIX97029.1	*Monochamus alternatus*	206	7e-62	97
OBP8	c20567.graph_c0	453	131	YES	1-19	4.37	11.07	odorant-binding protein 18	AIX97033.1	*Monochamus alternatus*	128	2e-35	48
OBP9	c19295.graph_c0	689	142	YES	1-19	898.43	841.35	odorant binding protein 14	ARH65469.1	*Anoplophora glabripennis*	284	1e-95	97
OBP10	c1579.graph_c0	325	101	YES	ND	0.54	0.46	odorant-binding protein 9	AJM71483.1	*Tenebrio molitor*	193	2e-61	81
OBP11	c24682.graph_c2	447	124	YES	ND	17077.57	6557.42	odorant-binding protein 2	AHA39267.1	*Monochamus alternatus*	227	1e74	90
OBP12	c27165.graph_c0	525	129	YES	1-20	232.58	259.47	odorant binding protein 11	ARH65466.1	*Anoplophora glabripennis*	241	7e-80	92
OBP13	c27224.graph_c0	621	149	YES	1-16	23.63	14.15	odorant-binding protein 2	ALW95359.1	*Cryptolaemus montrouzieri*	139	8e-39	42
OBP14	c18867.graph_c0	202	32	YES	ND	25.37	0.74	odorant binding protein 7	ARH65462.1	*Anoplophora glabripennis*	112	3e-30	78
OBP15	c37526.graph_c0	218	61	YES	ND	1.07	0	odorant-binding protein 28	AIX97090.1	*Monochamus alternatus*	77.4	4e-17	54
OBP16	c13226.graph_c0	578	144	YES	1-21	5090.51	3125.33	minus-C odorant-binding protein 2	ADD70031.1	*Batocera horsfieldi*	164	1e-48	53
OBP17	c27345.graph_c0	943	133	NO	1-16	6453.25	1383.88	odorant-binding protein 5	AIX97020.1	*Monochamus alternatus*	246	3e-79	89
OBP18	c34712.graph_c0	1016	129	NO	1-19	64.07	57.61	odorant-binding protein 18	AIX97033.1	*Monochamus alternatus*	110	4e-26	45
OBP19	c29015.graph_c0	891	134	NO	1-16	53.78	46.75	odorant-binding protein 17	AIX97032.1	*Monochamus alternatus*	213	1e-66	86

OBP20	c25500.graph_c0	920	144	NO	1-21	44.30	16.62	odorant-binding protein 22	AIX97037.1	*Monochamus alternatus*	143	5e-39	48
OBP21	c8514.graph_c0	658	132	NO	1-17	202.47	65.34	odorant-binding protein 20	AIX97035.1	*Monochamus alternatus*	176	3e-53	68
OBP22	c28325.graph_c0	1376	178	NO	1-21	2.13	3.05	odorant-binding protein 25	AIX97019.1	*Monochamus alternatus*	337	2e-112	90
OBP23	c28678.graph_c0	609	140	YES	1-25	963.96	1087.68	odorant-binding protein 3	AHA39268.1	*Monochamus alternatus*	243	8e-80	83
OBP24	c29586.graph_c0	792	124	NO	1-18	178.48	228.63	odorant-binding protein 18	AIX97033.1	*Monochamus alternatus*	121	4e-31	49
OBP25	c24613.graph_c1	1185	142	YES	1-19	1196.25	177.50	odorant binding protein 7	ARH65462.1	*Anoplophora glabripennis*	278	1e-90	96
OBP26	c28535.graph_c1	1037	148	NO	1-25	433.31	79.47	odorant-binding protein 22	AIX97037.1	*Monochamus alternatus*	215	2e-66	69
OBP27	c24683.graph_c0	594	144	YES	1-21	227.13	22927.81	odorant binding protein 1	ARU83752.1	*Anoplophora glabripennis*	258	1e-85	94
OBP28	c28852.graph_c0	873	218	NO	1-18	101.65	101.11	odorant-binding protein 19	AIX97034.1	*Monochamus alternatus*	315	6e-106	81
OBP29	c28838.graph_c0	785	133	NO	1-18	153.69	249.36	odorant-binding protein	AHE13799.1	*Lissorhoptrus oryzophilus*	112	8e-28	43
OBP30	c25065.graph_c0	718	135	YES	1-17	17.19	23.30	odorant-binding protein 21	AIX97067.1	*Dastarcus helophoroides*	125	4e-33	49
OBP31	c24706.graph_c0	686	142	NO	1-18	11.71	11.72	odorant-binding protein 4	ALR72492.1	*Colaphellus bowringi*	110	2e-27	47
OBP32	c25924.graph_c0	598	132	YES	1-19	64.17	141.04	odorant-binding protein 7	AIX97022.1	*Monochamus alternatus*	187	3e-58	64
OBP33	c8550.graph_c0	623	137	YES	1-19	204.22	134.43	odorant-binding protein 27	AIX97089.1	*Monochamus alternatus*	137	6e-38	49
OBP34	c28613.graph_c0	2415	136	NO	1-22	21.42	7.86	odorant-binding protein 15	AIX97030.1	*Monochamus alternatus*	166	2e-44	61
OBP35	c46641.graph_c0	235	47	YES	1-37	0.99	0.00	odorant binding protein 6	ARH65461.1	*Anoplophora glabripennis*	139	6e-41	100
OBP36	c18594.graph_c0	498	116	YES	1-20	7.60	13.36	odorant-binding protein 14	ALR72502.1	*Colaphellus bowringi*	77.0	4e-15	31`
OBP37	c19304.graph_c0	713	136	YES	ND	932.63	986.33	odorant-binding protein 21	AIX97067.1	*Dastarcus helophoroides*	151	4e-43	61
OBP38	c27891.graph_c0	3544	136	NO	1-16	1072.67	651.92	odorant-binding protein 6	AJO67868.1	*Monochamus alternatus*	257	3e-76	92
OBP39	c22400.graph_c0	527	144	YES	1-21	1558.56	50.49	odorant-binding protein 1	ABR53888.1	*Monochamus alternatus*	155	1e-45	52
OBP40	c26340.graph_c0	1886	134	NO	1-16	937.18	488.31	odorant-binding protein 11	AIX97026.1	*Monochamus alternatus*	254	1e-78	90
OBP41	c26089.graph_c0	1047	148	YES	1-19	601.58	162.44	odorant-binding protein 8	AIX97023.1	*Monochamus alternatus*	188	7e-56	68
OBP42	c26551.graph_c0	480	126	YES	1-20	221.02	252.87	odorant binding protein 13	ARH65468.1	*Anoplophora glabripennis*	73	7e-42	88
OBP43	c28512.graph_c0	543	143	YES	1-22	60.91	53.30	odorant-binding protein 16	AIX97031.1	*Monochamus alternatus*	247	8e-82	80
OBP44	c27470.graph_c0	636	137	YES	1-18	780.40	747.47	odorant binding protein 12	ARH65467.1	*Anoplophora glabripennis*	281	1e-94	99

supplementary Table S4. Best BLASTX matches of *A. chinensis* putative OR genes.

Number	Gene ID	Unigene Length (bp)	ORF Length (AA)	Complete ORF	TM Number	FPKM		Blastx match					
						F	M	Name	Acc. number	Species	Score	E-value	Identity (%)
OR1	c45787.graph_c0	328	101	YES	2	0.00	0.45	odorant receptor OR34	ALR72577.1	*Colaphellus bowringi*	126	2e-32	55
OR2	c20217.graph_c0	431	107	YES	2	1.89	0.35	odorant receptor 60	EEZ99415.2	*Tribolium castaneum*	102	2e-23	42
OR3	c24928.graph_c0	912	152	NO	3	1.07	0.97	odorant receptor 26	APC94330.1	*Pyrrhalta aenescens*	310	3e-100	61
OR4	c565.graph_c0	257	58	YES	0	0.00	0.00	olfactory receptor 5	AIX97096.1	*Monochamus alternatus*	70.5	6e-14	82
OR5	c41958.graph_c0	427	78	YES	0	0.27	0.58	olfactory receptor 3	AIX97094.1	*Monochamus alternatus*	117	9e-31	65
OR6	c50204.graph_c0	394	86	YES	0	0.00	0.50	odorant receptor	XP_021926083.1	*Zootermopsis nevadensis*	77.4	4e-14	36
OR7	c713.graph_c0	404	62	YES	1	0.50	0.57	odorant receptor 17	APC94228.1	*Pyrrhalta maculicollis*	41.6	0.16	29
OR8	c40656.graph_c0	284	92	YES	2	0.62	0.52	odorant receptor OR37	ALR72580.1	*Colaphellus bowringi*	83.2	8e-17	42
OR9	c28561.graph_c0	1542	422	YES	6	2.25	2.23	odorant receptor OR26	ALR72569.1	*Colaphellus bowringi*	311	2e-98	43
OR10	c19674.graph_c0	619	148	YES	3	1.25	0.87	odorant receptor OR6	ALR72551.1	*Colaphellus bowringi*	155	2e-42	44
OR11	c20682.graph_c0	1474	435	YES	7	1.19	1.11	olfactory receptor 14	CAM84012.1	*Tribolium castaneum*	313	7e-99	42
OR12	c47453.graph_c0	393	45	YES	0	0.00	0.51	odorant receptor OR36	ALR72579.1	*Colaphellus bowringi*	134	4e-35	80
OR13	c4026.graph_c0	454	130	YES	3	0.51	0.44	odorant receptor OR36	ALR72579.1	*Colaphellus bowringi*	159	9e-45	57
OR14	c26040.graph_c0	994	244	NO	4	1.59	1.25	odorant receptor OR9	ALR72554.1	*Colaphellus bowringi*	171	7e-47	37
OR15	c35929.graph_c0	1218	385	YES	6	4.21	2.53	olfactory receptor	XP_001864544.1	*Culex quinquefasciatus*	69.7	2e-09	25
OR16	c9432.graph_c0	624	97	NO	1	0.75	0.80	odorant receptor OR20	ALR72565.1	*Colaphellus bowringi*	87	3e-17	26
OR17	c32307.graph_c0	1386	388	YES	5	3.51	1.50	odorant receptor 8	APC94315.1	*Pyrrhalta aenescens*	182	6e-49	30
OR18	c46193.graph_c0	330	103	YES	1	0.18	0.75	odorant receptor OR40	ALR72583.1	*Colaphellus bowringi*	75.9	8e-14	33
OR19	c11832.graph_c0	624	173	YES	2	1.33	2.18	olfactory receptor 3	AIX97138.1	*Rhyzopertha dominica*	83.2	3e-16	29
OR20	c42738.graph_c0	611	157	NO	1	0.76	0.08	odorant receptor 18	APC94230.1	*Pyrrhalta maculicollis*	149	8e-40	47
OR21	c18731.graph_c0	250	0	NO	NO	1.63	0.40	odorant receptor	AID61207.1	*Calliphora stygia*	47.8	2e-04	37

OR22	c40923.graph_c0	320	41	YES	1	0.91	0.31	odorant receptor OR26	ALR72569.1	*Colaphellus bowringi*	104	1e-24	60
OR23	c21660.graph_c0	570	167	YES	3	0.41	0.35	odorant receptor OR40	ALR72583.1	*Colaphellus bowringi*	111	6e-26	37
OR24	c31668.graph_c0	1846	385	NO	7	1.62	1.69	odorant receptor OR6	ALR72551.1	*Colaphellus bowringi*	308	8e-96	40
OR25	c10861.graph_c0	684	160	YES	3	0.77	1.23	odorant receptor OR26	ALR72569.1	*Colaphellus bowringi*	196	7e-58	50
OR26	c20001.graph_c0	969	197	NO	2	0.71	1.59	odorant receptor 10	KFB39838.1	*Anopheles sinensis*	104	6e-22	27
OR27	c31026.graph_c0	1662	394	NO	5	1.68	1.72	odorant receptor OR9	ALR72554.1	*Colaphellus bowringi*	203	5e-57	35
OR28	c37060.graph_c0	1283	260	NO	4	5.16	3.97	odorant receptor OR40	ALR72583.1	*Colaphellus bowringi*	148	6e-37	31
OR39	c27905.graph_c0	834	275	YES	4	3.16	1.20	odorant receptor OR26	ALR72569.1	*Colaphellus bowringi*	192	2e-55	39
OR30	c43209.graph_c0	478	99	YES	2	0.49	0.42	odorant receptor 102	EEZ97750.2	*Tribolium castaneum*	114	3e-27	44
OR31	c33237.graph_c0	1481	380	YES	4	2.33	3.98	odorant receptor OR28	ALR72571.1	*Colaphellus bowringi*	168	8e-44	31
OR32	c31236.graph_c0	977	203	YES	3	2.44	1.78	odorant receptor 60	EEZ99415.2	*Tribolium castaneum*	93.6	4e-18	30
OR33	c29981.graph_c0	1274	396	YES	5	1.73	1.66	odorant receptor OR3	ALR72548.1	*Colaphellus bowringi*	312	1e-100	51
OR34	c27458.graph_c0	545	154	YES	3	0.53	1.91	odorant receptor OR24	ALR72568.1	*Colaphellus bowringi*	199	3e-59	49
OR35	c35376.graph_c0	2247	464	NO	7	48.35	54.90	olfactory receptor 1	AIX97092.1	*Monochamus alternatus*	908	0.0	97
OR36	c56834.graph_c0	220	39	YES	0	0.00	0.23	odorant receptor OR3	ALR72548.1	*Colaphellus bowringi*	77.4	4e-15	52
OR37	c28357.graph_c0	1464	368	YES	6	0.85	1.44	odorant receptor OR32	ALR72575.1	*Colaphellus bowringi*	210	7e-60	30
OR38	c27308.graph_c0	1464	384	YES	7	2.27	2.54	odorant receptor OR17	ALR72562.1	*Colaphellus bowringi*	342	1e-110	44
OR39	c18731.graph_c1	275	75	YES	0	0.00	0.36	olfactory receptor 3	AIX97138.1	*Rhyzopertha dominica*	58.9	1e-08	33
OR40	c39937.graph_c0	351	115	YES	0	0.17	0.99	odorant receptor OR40	ALR72583.1	*Colaphellus bowringi*	82.8	3e-16	32
OR41	c23797.graph_c0	1433	385	YES	7	2.03	0.76	odorant receptor OR6	ALR72551.1	*Colaphellus bowringi*	276	4e-85	40
OR42	c18726.graph_c0	229	53	YES	0	0.00	0.43	Odorant receptor 33a-like protein	KYB29103.1	*Tribolium castaneum*	107	7e-27	63
OR43	c25521.graph_c0	1153	328	YES	5	2.58	1.33	olfactory receptor OR16	AJO62235.1	*Tenebrio molitor*	274	7e-86	42
OR44	c15762.graph_c0	990	274	YES	2	0.59	0.90	odorant receptor 4	APC94309.1	*Pyrrhalta aenescens*	218	4e-64	39

supplementary Table S5. Best BLASTX matches of *A. chinensis* putative IR and SNMP genes.

Number	Gene ID	Unigene Length (bp)	ORF Length (AA)	Complete ORF	TM Number	FPKM		Blastx match					
						F	M	Name	Acc. number	Species	Score	E-value	Identity (%)
IR1	c30828.graph_c0	1371	386	YES	2	1.75	0.48	ionotropic receptor 3	APC94260.1	*Pyrrhalta maculicollis*	649	0.00	91
IR2	c35403.graph_c0	3429	842	NO	3	5.86	4.80	ionotropic receptor 8	APC94353.1	*Pyrrhalta aenescens*	999	0.00	63
IR3	c18911.graph_c0	2488	552	NO	3	28.32	6.78	ionotropic receptor onIR93a.1	J74495.1	*Dendroctonus ponderosae*	610	0.00	60
IR4	c12617.graph_c0	385	126	YES	1	11.04	10.96	ionotropic receptor 1	ANQ46493.1	*Phyllotreta striolata*	160	4e-43	61
IR5	c29334.graph_c0	612	138	YES	1	8.66	8.03	ionotropic receptor 1	ANQ46493.1	*Phyllotreta striolata*	290	3e-89	65
IR6	c36067.graph_c0	1978	567	YES	3	23.82	13.52	ionotropic receptor 6	ANQ46498.1	*Phyllotreta striolata*	388	3e-120	38
IR7	c23496.graph_c0	767	146	YES	0	0.84	1.04	ionotropic receptor 8a	ALR72538.1	*Colaphellus bowringi*	286	1e-87	53
IR8	c33610.graph_c0	3002	794	NO	4	2.37	3.10	ionotropic receptor 4	ANQ46496.1	*Phyllotreta striolata*	1584	0.00	87
IR9	c35007.graph_c0	3047	920	YES	3	3.86	9.76	ionotropic receptor 7	APC94352.1	*Pyrrhalta aenescens*	1045	0.00	61
IR10	c35544.graph_c0	2928	911	YES	3	81.78	101.72	ionotropic receptor 1	ANQ46493.1	*Phyllotreta striolata*	999	0.00	58
IR11	c34251.graph_c0	2605	745	NO	4	2.26	2.10	ionotropic receptor IR6	ALR72535.1	*Colaphellus bowringi*	1196	0.00	83
IR12	c36276.graph_c0	2669	539	NO	3	2.60	3.90	ionotropic receptor x	AKC58590.1	*Anomala corpulenta*	614	0.00	57
IR13	c13169.graph_c0	728	165	YES	1	0.64	0.41	ionotropic receptor 7	ANQ46499.1	*Phyllotreta striolata*	364	4e-117	72
IR14	c30828.graph_c0	1371	386	YES	0	1.75	0.48	ionotropic receptor 3	APC94260.1	*Pyrrhalta maculicollis*	649	0.00	91
IR15	c25729.graph_c0	1010	271	YES	1	0.81	1.38	ionotropic receptor 8a	ALR72538.1	*Colaphellus bowringi*	572	0.00	86
IR16	c38420.graph_c0	332	82	YES	1	0.88	0.15	ionotropic receptor 4	AIX97160.1	*Monochamus alternatus*	178	7e-55	91
IR17	c32049.graph_c0	2213	577	NO	3	14.29	9.47	ionotropic receptor 1	APC94347.1	*Pyrrhalta aenescens*	615	0.00	55
IR18	c1782.graph_c0	913	302	YES	2	0.00	1.09	ionotropic receptor IR2	ALR72541.1	*Colaphellus bowringi*	452	6e-54	71
IR19	c34317.graph_c0	2967	936	YES	3	29.46	31.44	ionotropic receptor 7	APC94261.1	*Pyrrhalta maculicollis*	946	0.00	56
IR20	c33610.graph_c0	3002	794	NO	4	2.37	3.10	ionotropic receptor 4	ANQ46496.1	*Phyllotreta striolata*	1584	0.00	87
IR21	c41683.graph_c0	202	0	NO	0	0.58	0.74	ionotropic receptor IR5	ALR72540.1	*Colaphellus bowringi*	88.2	7e-19	62

IR22	c4277.graph_c0	405	121	YES	1	0.00	0.74	ionotropic receptor IR4	AJO62242.1	*Tenebrio molitor*	221	8e-68	77
IR23	c5144.graph_c0	490	115	YES	2	0.24	0.51	ionotropic receptor 4	APC94262.1	*Pyrrhalta maculicollis*	258	4e-79	70
SNMP1	c30293.graph_c0	1943	529	YES	2	7.8	2.41	sensory neuron membrane protein SNMP1b	ALR72543.1	*Colaphellus bowringi*	565	0.00	57
SNMP2	c28038.graph_c0	1072	283	NO	1	1.49	1.41	sensory neuron membrane protein SNMP3	ALR72545.1	*Colaphellus bowringi*	368	3e-121	58
SNMP3	c33255.graph_c0	3714	561	YES	1	426.32	556.28	sensory neuron membrane protein 1	AIX97076.1	*Monochamus alternatus*	890	0.00	87

supplementary Table S6. Best BLASTX matches of *A. chinensis* putative GR genes.

Number	Gene ID	Unigene Length (bp)	ORF Length (AA)	Complete ORF	TM Number	FPKM		Blastx match					
						F	M	Name	Acc. number	Species	Score	E-value	Identity (%)
GR1	c24154.graph_c0	550	151	YES	3	0.34	2.05	gustatory receptor candidate 58	CAL23191.2	*Tribolium castaneum*	62	4e-08	36
GR2	c10441.graph_c0	1298	357	YES	7	1.03	0.57	gustatory receptor 6	APC94342.1	*Pyrrhalta aenescens*	171	4e-45	32
GR3	c44854.graph_c0	229	33	YES	0	0.00	0.86	gustatory receptor candidate 58	CAL23191.2	*Tribolium castaneum*	46.6	4e-04	39
GR4	c23899.graph_c0	669	132	YES	0	0.82	2.57	gustatory receptor 2	NP_001161916.1	*Tribolium castaneum*	281	4e-91	63
GR5	c13404.graph_c0	340	39	YES	0	1.03	0.29	gustatory receptor 109	EFA07606.1	*Tribolium castaneum*	52.4	2e-05	38
GR6	c32904.graph_c0	578	388	YES	6	3.87	4.26	gustatory receptor 160	EFA12223.1	*Tribolium castaneum*	119	9e-26	27
GR7	c58291.graph_c0	205	52	YES	1	0.00	0.24	gustatory receptor PhGr6	XP_002426236.1	*Pediculus humanus corporis*	40.4	0.01	35
GR8	c47817.graph_c0	314	52	YES	1	0.37	0.00	gustatory receptor 12	EFA04718.1	*Tribolium castaneum*	70.9	4e-12	43
GR9	c11922.graph_c0	505	145	YES	1	0.62	1.27	gustatory receptor 43a	KOC67773.1	*Habropoda laboriosa*	110	2e-25	36
GR10	c43046.graph_c0	359	117	YES	2	0.65	0.28	gustatory receptor 7	APC94254.1	*Pyrrhalta maculicollis*	182	2e-74	68
GR11	c28760.graph_c0	1542	412	YES	7	4.68	0.09	gustatory receptor 2	ALR72528.1	*Colaphellus bowringi*	330	2e-107	58
GR12	c21625.graph_c0	554	73	NO	1	1.06	0.63	gustatory receptor GR10	ALR72587.1	*Colaphellus bowringi*	66.6	2e-11	41

GR13	c6784.graph_c0	369	117	YES	2	0.00	0.67	gustatory receptor 153	EFA07631.2	*Tribolium castaneum*	71.6	3e-12	46
GR14	c7683.graph_c0	305	82	YES	1	0.00	0.49	gustatory receptor 102	EFA02935.1	*Tribolium castaneum*	78.2	6e-15	48
GR15	c66696.graph_c0	245	NO	NO	0	0.24	0.21	glutamate receptor 2.7	KHN42943.1	*Glycine soja*	89.7	3e-19	93
GR16	c24913.graph_c0	830	217	NO	2	0.58	0.93	gustatory receptor candidate 55	CAL23188.2	*Tribolium castaneum*	219	2e-65	52
GR17	c43047.graph_c0	729	231	YES	0	0.40	0.20	glutamate receptor 1	KOX67415.1	*Melipona quadrifasciata*	372	5e-121	73
GR18	c47618.graph_c0	214	62	YES	0	0.00	0.23	glutamate receptor 1	KZC05526.1	*Dufourea novaeangliae*	134	6e-35	90
GR19	c24242.graph_c0	2216	652	NO	3	7.49	0.17	Glutamate receptor	KYB24694.1	*Tribolium castaneum*	395	1e-127	53

supplementary Table S7. Sequences comparison between *A. chinensis* and *A. glabripennis*

**Name1**	***A. chinensis* ID**	**Name2**	***A.glabripennis* ID**	**Identity(%)**	**Alignment length**	**Mismatches**	**Gap**	**E-value**	**Bit Score**
AchiCSP1	c8588.graph_c0	AglaCSP5	Unigene5322	96.75	400	13	0	0.00E+00	690
AchiCSP2	c14414.graph_c0	AglaCSP11	Unigene12790	95.74	962	24	2	0.00E+00	1576
AchiCSP3	c26429.graph_c0	AglaCSP10	Unigene4446	95.3	830	25	2	0.00E+00	1344
AchiCSP7	c26587.graph_c0	AglaCSP12	Unigene10121	94.76	764	29	2	0.00E+00	1199
AchiCSP8	c19256.graph_c0	AglaCSP1	Unigene16278	95.56	806	20	1	0.00E+00	1020
AchiCSP9	c13555.graph_c0	AglaCSP4	Unigene5579	97	533	14	1	0.00E+00	924
AchiCSP10	c24670.graph_c0	AglaCSP9	Unigene9546	97.94	582	12	0	0.00E+00	1059
AchiCSP11	c13227.graph_c0	AglaCSP2	Unigene6655	96.76	740	16	2	0.00E+00	1273
AchiCSP12	c29096.graph_c0	AglaCSP7	Unigene11951	96.39	776	23	2	0.00E+00	1306
AchiCSP13	c30244.graph_c0	AglaCSP3	Unigene5172	97.08	479	14	0	0.00E+00	839
AchiCSP15	c34532.graph_c0	AglaCSP8	Unigene14677	96.65	1105	24	1	0.00E+00	1893
AchiCSP16	c8520.graph_c0	AglaCSP6	Unigene5135	95.68	533	22	1	0.00E+00	866

supplementary Table S7. Primers for fluorescence quantitative real-time PCR.

Name	Forward primer	Reverse primer
*Achi*CSP1	GGACAGGGGTGTAGCA	ACCAGTTCGTTCCACCACTC
*Achi*CSP2	TTGTGACCAGGCGTGG	CGTGCTTCGCGGGT
C*Achi*SP3	TCGTGCTTGATGGACGAG	CCAGTCTTTGGCTCTCTTCG
*Achi*CSP4	TTGTGCCTTTCCGTGGTTAT	GCACCGTTTCTCTGCTTTTC
*Achi*CSP5	TCAGGTAGCCCTTTTGCTGT	ACGACCTACGGGATCACG
*Achi*CSP6	TTCGACTGTGGATCTGGA	CTCGGCATTTTTCGCATT
*Achi*CSP7	TATGACCCAGGGCAGC	CCAGATTGGCCTGATTGTCT
*Achi*CSP8	TGCCGACGATATACACCA	TCGCTGCATTTTGCAGTC
*Achi*CSP9	TTGACGCCGTCATTGTTA	GGCCTCAGGGACATGATCT
*Achi*CSP10	GGCATACGTTTTAGCCGTGT	TTTCTTGGTGCCGTCTTTCT
*Achi*CSP11	CGCCATCGATTGTGTG	ACGTCGATCCCCTCCTTT
*Achi*CSP12	CATCGACTGTGACGGAGA	ATTTGCTGCAGTCCGTTTCT
*Achi*CSP13	CTCTTTGCGCCTTTGCTTAC	CATTGCACTGGGCATTTG
*Achi*CSP14	ACGTTTGCATGCGTTGTA	CATCACCCACGACCAC
*Achi*CSP15	CACCCGTCGCCACT	CCTGTTTCCCACCCTGTT
*Achi*CSP16	ATTCCCGATGCACTTTTGAC	CCTTCTTTGGCGCTCTTG
*Achi*PBP1	TGGTGGACATGCTTCACT	ATGGTCGCCTCCACATCTAC
*Achi*PBP2	GCTTAGCCAGCACTGGAGTC	CGACCCGTATATGGTTCCAC
GAPDH	ACATCGGAGACTCTGCTACG	CTTCATGATGGAGTTGTAGGTGGT

supplementary Table S8. Protein numbers and gene accession numbers used in phylogenetic trees.

OBP		CSP		OR	
Number	GI	Number	GI	Number	GI
TcasOBP1	270009230	TcasCSP1	112031645	TcasOR1	91084439
TcasOBP2	1004398999	TcasCSP2	113951657	TcasOR2	642937874
TcasOBP3	270009227	TcasCSP3	113031988	TcasOR3	642935960
TcasOBP4	270009294	TcasCSP4	113951783	TcasOR4	642935272
TcasOBP5	270009229	TcasCSP5	113951798	TcasOR5	642925912
TcasOBP6	270008146	TcasCSP6	113951719	TcasOR6	189241169
TcasOBP7	270008145	TcasCSP7	113951740	TcasOR7	642937146
TcasOBP8	1004399517	TcasCSP8	113951758	TcasOR8	642935270
TcasOBP9	270014265	TcasCSP9	113951779	TcasOR9	189235390
TcasOBP10	270011094	TcasCSP10	113951696	TcasOR10	642912833
TcasOBP11	270009247	TcasCSP11	112031814	TcasOR11	642912741
TcasOBP12	270006409	TcasCSP12	112031836	TcasOR12	642937340
TcasOBP13	270006410	TcasCSP13	112031855	TcasOR13	642935746
TcasOBP14	270006466	TcasCSP14	112031878	TcasOR14	642933841
TcasOBP15	270015618	TcasCSP15	112031903	TcasOR15	642932549
TcasOBP16	1004400381	TcasCSP6	112031918	TcasOR16	642932545
TcasOBP17	270006413	TcasCSP17	112031943	TcasOR17	642932541
TcasOBP18	270006412	TmolCSP1	758213788	TcasOR18	642932539
TcasOBP19	270006512	TmolCSP2	758213790	TcasOR19	642934097
TcasOBP20	1004399000	TmolCSP3	758213792	TcasOR20	642925430
TcasOBP21	1004397441	TmolCSP4	758213794	TcasOR21	642915440
TcasOBP22	1004396940	TmolCSP5	124246517	TcasOR22	642915438
TcasOBP23	270014355	TmolCSP6	758213798	TcasOR23	642915016
TcasOBP24	270008128	TmolCSP7	758213800	TcasOR24	642915133
TcasOBP25	1004399686	TmolCSP8	758213802	TcasOR25	642914696
TcasOBP26	1004399687	TmolCSP9	758213804	TcasOR26	189236824
TcasOBP-C01	270011096	TmolCSP10	758213806	TcasOR27	91091874
TcasOBP-C02	270011097	TmolCSP11	758213808	TcasOR28	642912743
TcasOBP-C03	270011098	TmolCSP12	758213810	TcasOR29	642937148
TcasOBP-C04	270010982	MaltCSP1	723456379	TcasOR30	91084439
TcasOBP-C05	270011095	MaltCSP2	723456381	TcasOR31	642920568
TcasOBP-C06	270011100	MaltCSP3	723456383	TcasOR32	91089003
TcasOBP-C07	270011099	MaltCSP4	723456385	TcasOR33	91091912
TcasOBP-C08	270011190	MaltCSP5	723456387	DponOR1	471180445
TcasOBP-C09	270010981	MaltCSP6	723456389	DponOR2	471180431
TcasOBP-C10	270001294	MaltCSP7	723456391	DponOR3	459442396
TcasOBP-C11	270001292	MaltCSP8	723456377	DponOR4	459442392
TcasOBP-C12	270001293	MaltCSP9	723456465	DponOR6	459442380
TcasOBP-C13	270001342	MaltCSP11	723456469	DponOR8	459442370
TcasOBP-C14	270011070	DponCSP1	471180421	DponOR10	459442362

TcasOBP-C15	270006378	DponCSP2	471180443	DponOR11	459442358
TcasOBP-C16	270006441	DponCSP3	471180419	DponOR12	459442356
TcasOBP-C17	270006442	DponCSP4	828177651	DponOR13	459442348
TcasOBP-C18	270002750	DponCSP6	471180423	DponOR15	459442342
TcasOBP-C19	270014235	DponCSP8	471180427	DponOR17	459442334
TcasOBP-C20	270004977	ItypCSP1	459277247	DponOR18	459442332
TcasOBP-C21	270011043	ItypCSP2	459277243	DponOR19	459442328
DmelOBP19c	268056755	ItypCSP3	459277241	DponOR21	459442318
DmelOBP83b	24644477	**GR**		DponOR22	459442316
DmelOBP83e	24644507	Number	GI	DponOR23	459442312
DmelOBP83g	24644509	TcasGR1	1004397801	DponOR25	459442300
DmelOBP50a	45552617	TcasGR2	270297167	DponOR26	459442298
DmelOBP50b	24653633	TcasGR3	224458340	DponOR27	459442292
DmelOBP50c	665400904	TcasGR9	125629112	DponOR28	459442294
DmelOBP50d	45551098	TcasGR10	224458354	DponOR29	459442290
DmelOBP50e	45550427	TcasGR11	125629116	DponOR30	459442288
DmelOBP56a	19922608	TcasGR15	125629124	DponOR32	459442284
DmelOBP56b	24655939	TcasGR17	125629128	DponOR33	459442282
DmelOBP56c	158939314	TcasGR18	125629130	DponOR34	459442280
DmelOBP56d	158703566	TcasGR19	270008277	DponOR35	459442384
DmelOBP56e	158703666	TcasGR20	299523140	DponOR36	459442372
DmelOBP56f	24655956	TcasGR21	299523149	DponOR38	459442368
DmelOBP56g	158703866	TcasGR25	270017141	DponOR39	459442336
DmelOBP56h	158703966	TcasGR26	1004404015	DponOR40	459442320
DmelOBP56i	158704032	TcasGR28	270009313	DponOR41	459442286
DmelOBP49a	24653178	TcasGR30	1004402339	ItypOR1	459277392
DmelOBP47a	968087174	TcasGR32	270009314	ItypOR2	459277390
DmelOBP47b	24652686	TcasGR35	125629063	ItypOR3	459277388
DmelOBP58b	24658429	TcasGR37	270012835	ItypOR4	459277386
DmelOBP58c	7291466	TcasGR39	270009317	ItypOR5	459277384
DmelOBP58d	24658441	TcasGR41	125629075	ItypOR6	459277382
DmelOBP85a	1114645773	TcasGR45	1004399181	ItypOR7	459277380
DmelOBP57a	24656247	TcasGR46	125629033	ItypOR8	459277378
DmelOBP57c	19922636	TcasGR47	270009324	ItypOR9	459277374
DmelOBP57d	24656292	TcasGR49	270009326	ItypOR10	459277372
DmelOBP57e	22026979	TcasGR50	270009327	ItypOR11	459277370
DmelOBP93a	24648633	TcasGR52	1004399211	ItypOR12	459277368
DponOBP1	828177608	TcasGR56	125629049	ItypOR13	459277366
DponOBP2	828177610	TcasGR57	270011151	ItypOR14	459277364
DponOBP6	828177618	TcasGR60	270011154	ItypOR15	459277362
DponOBP9	828177622	TcasGR62	157885913	ItypOR16	459277360
DponOBP10	828177624	TcasGR65	157885911	ItypOR17	459277358
DponOBP12	828177627	TcasGR67	270009330	ItypOR18	459277356
DponOBP13	828177629	TcasGR72	270015367	ItypOR19	459277354

DponOBP16	828177634	TcasGR73	270002957	ItypOR20	459277352
DponOBP17	828177636	TcasGR87	270002937	ItypOR21	459277350
DponOBP18	828177638	TcasGR89	270016254	ItypOR22	459277348
DponOBP19	828177640	TcasGR91	270016253	ItypOR23	459277346
DponOBP21	828177644	TcasGR92	270015728	ItypOR24	459277344
DponOBP22	385200018	TcasGR107	270011156	ItypOR25	459277341
DponOBP24	385200020	TcasGR108	224458352	ItypOR26	459277339
DponOBP25	385200022	TcasGR117	270011166	ItypOR27	459277337
DponOBP26	385200024	TcasGR118	270011167	ItypOR28	459277335
ItypOBP2	459277271	TcasGR121	270011170	ItypOR33	459277327
ItypOBP3	459277269	TcasGR143	270001321	BmorOR1	112983558
ItypOBP4	459277267	TcasGR144	1004395364	BmorOR2	112983084
ItypOBP5	459277263	TcasGR154	270011184	BmorOR3	112982950
ItypOBP6	459277261	TcasGR155	270011185	BmorOR4	112982926
ItypOBP10	459277253	4594423160	270015775	BmorOR5	112982948
ItypOBP12	459277273	TcasGR164	270002945	BmorOR6	112982988
BhorPBP1	701219148	TcasGR204	270011189	BmorOR7	163838688
BhorPBP2	701219150	ItypGR1	459277307	BmorOR8	254939543
HparPBP1	295291564	ItypGR2	459277305	BmorOR9	182509188
HparPBP2	295291594	ItypGR3	459277303	BmorOR11	290563295
ItypOBP12	459277273	DponGR1	459442338	BmorOR12	162462524
**IR**		DponGR2	459442296	BmorOR13	290559921
Number	ID	DmelGR64a	24657115	BmorOR14	290560836
TmolIR1	758213852	DmelGR64b	24657120	BmorOR15	148298756
TmolIR2	758213854	DmelGR64c	23092959	BmorOR16	290566751
TmolIR4	758213858	DmelGR64d	665409876	BmorOR18	290651022
TmolIR5	758213860	DmelGR64e	78711776	BmorOR19	148298665
TmolIR6	758213862	DmelGR64f	45551511	BmorOR22	290560841
DponIR8a	471180437	DmelGR5a	24639922	BmorOR34	158711753
DponIR76b	459442376	DmelGR61a	440215090	BmorOR35	158508574
DponIR75q	459442322	DmelGR63a	221330835	BmorOR36	290650661
DponIR25a	459442302	DmelGR21a	118500892	BmorOR41	148298822
ItypIR25a	459277285	DmelGR92a	45551934	BmorOR44	290563344
DmelIR93a	442620186	DmelGR89a	45550757	BmorOR45	162461258
DmelIR25a	316994955	DmelGR93a	24648814	BmorOR46	240255410
DmelIR76a	281366466	DmelGR93b	221458282	BmorOR47	162462595
DmelIR76b	24667182	DmelGR93c	24648820	BmorOR50	197914319
DmelIR94b	442620405	DmelGR93d	24648822	BmorOR54	290560855
DmelIR94a	24648907	DmelGR58a	45551148	BmorOR61	290560861
DmelIR76a	281366466	DmelGR58b	28573670	BmorOR63	290563364
DmelIR76b	24667182	DmelGR36a	45550994	DmelOR1	24644231
DmelIR52a	221330289	DmelGR36b	24584786	DmelOR2	17647761
DmelIR52b	221330291	DmelGR36c	24584788	DmelOR3	45552543
DmelIR52c	24653873	DmelGR59a	45551154	DmelOR4	24586190

DmelIR52d	221330293
DmelIR56a	24655429
DmelIR56b	24655838
DmelIR56c	24655843
DmelIR56d	24655848
DmelIR67a	442631348
DmelIR67b	24662201
DmelIR67c	24662205
DmelIR7a	24640399
DmelIR7b	442615429
DmelIR7c	386764010
DmelIR7d	221329764
DmelIR7e	221329766
DmelIR7f	221329768
DmelIR7g	221329770
DmelIR75a	221512773
DmelIR75b	281366389
DmelIR75c	281366391
DmelIR75d	386771401
DmelIR60a	24762594
DmelIR60b	221468675
DmelIR60d	281364203
DmelIR100a	45550870
DmelIR10a	161077734
DmelIR94a	24648907
DmelIR76a	281366466
DmelIR76b	24667182
DmelIR94b	442620405
DmelIR94a	24648907
TmolIR1	758213852
TmolIR2	758213854
TmolIR4	758213858
TmolIR5	758213860
TmolIR6	758213862
DponIR8a	471180437
DponIR76b	459442376
DponIR75q	459442322

DmelGR59b	24659066
DmelGR59c	24659098
DmelGR59d	45550499
DmelGR22a	24580943
DmelGR22b	62471407
DmelGR22c	45550917
DmelGR22d	442625250
DmelGR22e	24580939
DmelGR28a	45549155
DmelGR28b	45445043
DmelGR98a	24650636
DmelGR98b	24650628
DmelGR98c	24650638
DmelGR8a	28571153
DmelGR10a	24641287

SNMP	
Number	GI
TcasSNMP1	189236600
TcasSNMP2	91092044
DponSNMP1	471180467
DponSNMP2	471180441
ItypSNMP1	459277283
TmolSNMP1	758213866
TmolSNMP2	758213864
AglaSNMP1	550232442
AglaSNMP2	550247955
CbowSNMP1	959478458
CbowSNMP2	959478460

DmelOR5	22023972
DmelOR6	22023986
DmelOR7	386771133
DmelOR8	45550587
DmelOR9	45549172
DmelOR10	17737459
DmelOR11	17137662
DmelOR12	28574001
DmelOR13	17986023
DmelOR14	22023974
DmelOR15	24584456
DmelOR16	17933522
DmelOR17	17647783
DmelOR18	442617999
DmelOR19	24661763
DmelOR20	17647785
DmelOR21	24656307
DmelOR22	17647765
DmelOR23	17530847
DmelOR24	45553085
DmelOR25	17647767
DmelOR26	24643875
DmelOR27	24650735
DmelOR28	17737845
DmelOR29	24643445
DmelOR30	24659319
DmelOR31	24646756
DmelOR32	24648414
DmelOR33	24661721
DmelOR34	17647781
DmelOR35	17986019
DmelOR36	24655965
DmelOR37	17986095
DmelOR38	17647763
DmelOR39	28573387
DmelOR40	17986171
DmelOR41	24662175
DmelOR42	17986079
DmelOR43	28573313
DmelOR44	24645077
DmelOR45	17986153
DmelOR46	24580998
DmelOR47	17986175
DmelOR48	17738135

DmelOR49	24638847
DmelOR50	24582997
DmelOR51	24644502
DmelOR52	24642365
DmelOR53	17738133
DmelOR54	221472522
DmelOR55	85816214
DmelOR56	85816212
DmelOR57	24644225
DmelOR58	78707529
DmelOR59	24643435
